# Dysbiotic Lesional Microbiome With Filaggrin Missense Variants Associate With Atopic Dermatitis in India

**DOI:** 10.3389/fcimb.2020.570423

**Published:** 2020-11-17

**Authors:** Shankha Nath, Naina Kumari, Debabrata Bandyopadhyay, Neloy Sinha, Partha P. Majumder, Rupak Mitra, Souvik Mukherjee

**Affiliations:** ^1^National Institute of Biomedical Genomics, Kalyani, India; ^2^Medical College and Hospital, Kolkata, India; ^3^College of Medicine and JNM Hospital, Kalyani, India; ^4^Indian Statistical Institute, Kolkata, India; ^5^Unilever R & D, Bangalore, India

**Keywords:** atopic dermatitis, skin microbiome, host-microbiome association, Filaggrin, *Staphylococcus aureus*, microbial pathway

## Abstract

**Background:** Atopic Dermatitis (AD) has been associated with the loss of function (LoF) mutations in Filaggrin (*FLG*) gene and increase in relative abundance of specific microbes in the lesional skin, predominantly in Caucasians. Our study aims to determine, in Indian AD patients, (a) the prevalence of *FLG* LoF and missense mutations, and (b) the nature and extent of dysbiosis and altered microbial pathways with and without mutations in *FLG*. AD patients (*n* = 34) and healthy controls (*n* = 54) were recruited from India in this study and shotgun sequencing was carried out in a subset of samples with adequate microbiome DNA concentration. Host DNA from the same subset of samples was subjected to *FLG* coding region sequencing and host-microbiome association was estimated.

**Results:** The prevalence of *FLG* LoFs that are associated with AD globally were significantly lesser in our cases and controls (8.6%, 0%) than those reported in Europeans (27%, 2.6%). *Staphylococcus aureus* was present only on AD skin [abundance in Pediatric AD: 32.86%; Adult AD: 22.17%], but not on healthy skin on which *Staphylococcus hominis* (Adult controls: 16.43%, Adult AD: 0.20%; *p* = 0.002), *Cutibacterium acne*s (Adult controls:10.84%, Adult AD: 0.90%; *p* = 0.02), and *Malassezia globosa* (Adult controls: 8.89%, Adult AD: 0.005%; *p* = 0.001) were significantly more abundant. Microbial pathways mostly associated with skin barrier permeability, ammonia production and inflammation (Arginine and Proline metabolism, Histidine Metabolism and *Staphylococcus aureus* infection) were significantly enriched on AD skin metagenome. These pathways are also reported to impair antimicrobial peptide activity. Among AD patients with missense single nucleotide polymorphisms harboring “potentially damaging” alleles in *FLG* gene, damaging allele dosage was significantly (*p* < 0.02) positively correlated with relative abundance of phylum_Proteobacteria up to order_*Pseudomonadales* and negatively correlated with phylum_*Firmicutes* up to species_*Staphylococcus aureus*.

**Conclusion:** Our study has provided evidence that host DNA profile is significantly associated with microbiome composition in the development of AD. Species and strain level analysis showed that the microbial pathways enriched in AD cases were mostly found in MRSA strains. These evidences can be harnessed to control AD by modulating the microbiome using a personalized strategy. Our findings on the association of *FLG* genotypes with the microbiome dysbiosis may pave the way for a personalized strategy to provide a more effective control of AD.

## Introduction

Atopic Dermatitis (AD) is a common, chronic dermatological disorder, associated with dry skin and characterized by persistent itching. AD affects about 20% of children and 10% of adults worldwide (Clausen et al., 2018). Variants in genes involved in maintenance of skin integrity (*FLG, ACTL9, SPINK5*) and inflammation (*TNFA, IL10, IL13*) have previously been found associated with AD (Kato et al., 2003; Bussmann et al., 2011; Lesiak et al., 2011; Paternoster et al., 2011; Behniafard et al., 2012; Portelli et al., 2015; Bauer, 2017; Kim and Leung, 2018). Loss-of-function (LoF) mutations in Filaggrin (*FLG*) gene, that encodes a protein responsible for maintaining skin barrier function, have been consistently found to be associated with AD. Interestingly, the sets of *FLG* LoF mutations predominantly associated with AD show great geographical variation and are disjoint among patients in Europe [R501X, 2282del4] (Sandilands et al., 2007), Asia [3321delA, S2554X] (Enomoto et al., 2008), and Africa [S3316X, R826X] (Winge et al., 2011; Margolis et al., 2018). This underscores the importance of screening *FLG* in AD patients in different geographical regions, including India. Filaggrin monomers are predominantly degraded into arginine and histidine amino acids that acts as natural moisturizing factors (NMFs) which maintain hydration and structural integrity in the *stratum corneum* (Candi et al., 2005; Miajlovic et al., 2010; Kobayashi and Nagao, 2019). NMFs can affect the keratinization process as well as microbial colonization by influencing skin surface pH. It has been reported that the presence of NMFs with acidic conditions in skin impede *S. aureus* growth (Miajlovic et al., 2010; Kobayashi and Nagao, 2019). Microbial species, such as, *S. aureus, S. haemolyticus, S. epidermidis*, and *Streptococcus* sp. were found to be associated with AD. Both microbiological culture and metagenomic sequencing have revealed significant dysbiosis and predominance of non-commensal microbiota (Dekio et al., 2007; Kong et al., 2012; Seite et al., 2014; Powers et al., 2015; Chng et al., 2016; Shi et al., 2016; Bjerre et al., 2017; Williams and Gallo, 2017) in the lesional skin of AD patients compared to healthy skin (Kong et al., 2012; Powers et al., 2015; Bjerre et al., 2017; Williams and Gallo, 2017). AD associated microbiome profiles are dissimilar among patients in USA [*S. aureus*] (Kong et al., 2012; Seite et al., 2014), Japan [*Staphylococcus* sp. and *Streptococcus* sp.] (Dekio et al., 2007) and Europe [*S. aureus, S. caprae, S. epidermidis*, and *Cutibacterium* sp.] (Clausen et al., 2018). Microbiome studies on AD patients from India are absent. Both host genetic factors and microbiome profiles in lesional skin have been independently associated with development of AD in previous studies (Dekio et al., 2007; Sandilands et al., 2007; Enomoto et al., 2008; Winge et al., 2011; Kong et al., 2012; Seite et al., 2014; Clausen et al., 2018; Margolis et al., 2018). Host-microbiome association in FLG deficient skin has been well-documented in Ichthyosis Vulgaris patients where Gram positive anaerobic cocci such as *Finegoldia, Anaerococcus*, and *Peptoniphilus* were found to be depleted. Recent studies on AD patients showed that individuals with *FLG* null mutations were characterized by overall reduction in microbial diversity (Chng et al., 2016) and higher abundance of *S. caprae* compared to those without the *FLG* mutations (Clausen et al., 2018). AD patients with higher abundance of *S. aureus* had been associated with upregulation of host genes involved in skin barrier function, tryptophan metabolism, immune activation, and T_H_2 signaling (Fyhrquist et al., 2019). However, the identification and association of specific microbes along with their gene families or functional pathways on the lesional skin of AD patients with and without *FLG* risk genotypes has not been done before (Ghosh et al., 2018). In this study, we have explored this and tested the hypothesis that AD patients carrying *FLG* risk genotypes will exhibit significantly altered microbiome profiles on lesional skin and enriched in distinct biosynthetic pathways compared to patients without the risk genotypes. Microbiome profiles in AD are known to vary with age, (Shi et al., 2016) therefore we included both pediatric and adult AD patients in our study. We have identified host-microbiome association in AD, and specific microbial pathways that are differentially abundant in lesional skin microbiome of AD patients and in skin of healthy controls. Our results provide insights on how microbes influence biology to cause dry skin.

## Materials and Methods

### Study Design and Sample Collection

The Institutional Ethics Committees of National Institute of Biomedical Genomics, Kalyani, and Medical College and Hospital, Kolkata, India, approved this study. Written, informed consent was obtained from all adult participants and from parents or guardians of pediatric patients. Patients were recruited from the Dermatology OPD of Medical College and Hospital following Williams Criteria (Williams et al., 1994) (Supplementary Table 1). Age and gender matched adult healthy controls with no previous report as well as family history of atopy or any other skin diseases were recruited in a ratio of 1:3 for Case: Control to adjust for inter-individual variability of skin microbiome in healthy individuals (The Human Microbiome Project et al., 2012). We have used prefix A, B, C for three age, gender, and site matched control samples for each AD cases. The disease severity was scored by measuring the EASI (Eczema Area and Severity Index) parameter in all the patients. Biospecimens—skin and lesional swabs, and 5 ml of both clotted and unclotted blood samples—were collected from an individual only if (s)he did not use any antibiotic for 2 weeks or any topical cream for 1 week prior to the day of biospecimen collection (The Human Microbiome Project et al., 2012). We collected samples from either antecubital fossa (elbow pit) or cervical (neck) region of the patients and controls. To account for the difference in sampling site, we have compared the predominant taxa (core microbiome) between the antecubital fossa (elbow pit) and the cervical (neck) region for patients and controls separately (Supplementary Table 2) before pooling them for further analysis. Site matched skin and lesional swabs were collected from controls and patients, respectively, using sterile cotton swab sticks in sterilized polypropylene tubes (HIMEDIA PW100) for both microbiome sequencing as well as quantitative cultures in universal media (Supplementary Material) (Klymiuk et al., 2016). For each patient or healthy control, serum IgE level was estimated by ELISA.

### Microbial DNA Isolation and Shotgun Sequencing

Microbial DNA was isolated from the healthy skin and lesional swab samples using MoBio Biostic Bacteremia Kit and the DNA concentration was quantified by Qubit Fluorometer. Metagenomic sequencing could only be performed on those samples with DNA concentration ≥0.2 ng/μl by Illumina HiSeq 2500 using Nextera XT kit following 2 × 250 bp paired-end chemistry (Robin et al., 2016).

### Metagenomic Data Analysis

Read pairs were aligned to the human genome (hg19) using Bowtie2 (v2.3.4.3) for identification and removal of human reads. Subsequently, read pairs with read length shorter than 36 bases and average quality value <25 were also removed using Trimmomatic tool of Kneaddata software (Bolger et al., 2014). Quality filtered reads were mapped by MetaphlAn2 to a database of clade specific marker genes comprising of 17,110 microbial genomes downloaded from Integrated Microbial Genome Archives (IMG/A) (Markowitz et al., 2011) to obtain a pan microbial profiling (Truong et al., 2015) which is then used by MetaPhlAn2 classifier for taxonomic classification from phylum to species (Segata et al., 2011).

### Functional Annotation of Metagenomes

Reads were mapped to ChocoPhlan pangenome database by HUMAnN2 (Abubucker et al., 2012) to quantify gene family abundance in RPK (Reads/Kilobase) for community total and species-resolved gene families. The RPK values were converted to Copies per Million (CPM) to account for sample sequencing depth. Unmapped reads were further mapped by translated search against a UniRef-based protein sequence database (UniRef-90) (Suzek et al., 2014). Finally, for gene families quantified at either the nucleotide or protein levels for each species in a sample, we used HUMAnN2 to reconstruct pathways and to estimate community total and species-resolved pathway abundances based on the KEGG (v56) pathway database (Karp et al., 2002).

### Identification of *S. aureus* by Quantitative Microbiological Culture

Quantitative microbiological culture was performed for estimating the colony forming units (CFU/ml) of aerobic culturable bacteria from lesional and healthy skin swabs collected from AD patients and healthy controls, respectively. Pure cultures were generated and Gram positive cocci were further tested for their Catalase and Coagulase activities (Barrow and Feltham, 1993).

### Blood DNA Isolation and *FLG* Gene Sequencing

Human DNA was isolated from unclotted blood using Qiagen Blood DNA Midi Kit following manufacturer's protocol. To identify LoF mutations and missense variants, the entire coding region (12.18 kb) of the *FLG* gene was sequenced using ABI-3500 Genetic Analyser in the same set of patients and controls among whom metagenomic sequencing could be performed. Chromatograms were analyzed using Seqscape v2.1.4. for identification of single nucleotide variations (SNVs) within the *FLG* coding sequence (CDS). Allele and genotype frequencies were estimated using PLINK v.1.9 (Purcell et al., 2007) and haplotypes were constructed using PHASE v2.1.1 (Stephens et al., 2001).

### Th1/Th-2-type Cytokine Assays From Serum Samples

Serum cytokine assays were performed using Human Cytokine Pro 27-Plex Assay kit that includes both Th1 and Th2 cytokines mostly associated with AD (Numerof and Asadullah, 2006). Only those adult AD patients with metagenomic data and one age-gender matched healthy control for each patient assayed using the BioPlex platform (Supplementary Material).

### Statistical Analyses

Differentially abundant microbial taxa between (a) adult patients and adult controls and (b) adult and pediatric patients were identified by Wilcoxon rank-sum test using the R command wilcox.test. Microbial taxa from phylum to species with mean relative abundance ≥1% and present in at least 50% of individuals in at least one group were termed as the “core microbiome” and included in all subsequent analysis. We have observed no significant difference in core microbiome while comparing the anatomical sites within patients and controls separately, hence we have pooled them for further analysis (Supplementary Table 2). Alpha diversity index (Shannon index) which accounts for species abundance and evenness were measured for all the patients and healthy controls using R package “vegan.” Nei's distance (Nei, 1972) between pairs of individuals was computed (Supplementary Material) from the species level taxonomic abundances. In computing Nei's distance measure, we have taken into account phylogenetic relationships among species comprising each microbial genus. The discriminant analysis as implemented on Lefse (Segata et al., 2011) was used to identify significantly discriminant microbial pathways between adult cases and controls. Discriminating pathways were further analyzed using species-resolved data for identification of distinct microbial species and/or strains harboring those pathways in patients and controls. Spearman's rank correlation was estimated between relative abundances of predominant microbial taxa from phylum to species levels and IgE or cytokine levels for patients and controls separately. IgE levels and cytokine expressions were compared between adult AD patients and healthy controls as well as between adult and pediatric cases. Disease severity index (EASI) values were correlated with proportions of predominant microbial taxa in both adult and pediatric cases. To validate the identification of the most abundant organism *S. aureus* in AD patients, relative abundances of *S. aureus* in microbiome data were correlated with the CFU/ml values obtained from quantitative cultures of its pure colonies.

*FLG* null and missense mutations were predicted as “potentially damaging” or “benign” by *in silico* based functional classification using Variant Effect Predictor (VEP) of Ensembl (Mclaren et al., 2016) For host-microbiome associations, the damaging allele dosages for each SNV was scored as AA = 0, Aa = 1, and aa = 2 (A= reference allele, a = mutant and damaging allele). For each patient or control, the damaging allele dosages were summed up for all the damaging SNVs. The correlation between damaging allele dosage and relative abundance of microbial taxa from phylum to species level were estimated among patients and controls separately. We compared the relative abundances of microbial pathways between individuals with reference allele homozygotes vs. mutant allele homozygotes for the damaging SNVs in cases and controls separately. The significant pathways were analyzed using species-resolved data for identifying specific microbes harboring those pathways.

## Results

### Characteristics of Study Participants

Thirty four AD patients, 18 adults (12 male, six female) and 16 children (eight male, eight female) and age and gender matched 54 adult healthy controls (1:3) were included. All patients (adults and children) had visible skin lesions predominantly in the inner side of the elbow (antecubital fossa) or neck (cervical spine) regions with characteristics of xerosis, keratosis pilaris and visible flexural dermatitis following Williams Criteria (Williams et al., 1994) (Supplementary Table 1). No difference in core microbiome composition between antecubital fossa (elbow pit) and cervical (neck) region was observed within patients and controls separately and hence these samples were pooled for further downstream analysis (Supplementary Table 2). The mean ages (in years) of the adult and pediatric patients and healthy controls were 34.8 ± 12.8 (Min–Max: 18–57), 8.8 ± 3.4 (Min–Max: 2–16), and 36.2 ± 12.7 (Min–Max: 18–57), respectively.

About 30% (10/34) of the patients had positive family history (affected first/second degree relatives), consistent with earlier studies in India (35.3%) (Sarkar and Kanwar, 2004), and Europe (22–26%) (Larsen, 1996). Most patients (33/34; 97.1%) presented with moderate (7 ≤ EASI ≤ 20; *n* = 21) to high (EASI>20; *n* = 12) disease severity scores with sleep disturbances throughout the year (15/21, 71%; 12/12, 100%). This is consistent with an earlier study from Europe where 89% of the AD patients showed sleep disturbances with moderate severity, and 100% with high severity (Sanchez-Perez et al., 2013). The average EASI score was higher in adults (24.1) than in pediatric patients (16.9); the difference was not statistically significant (*p* > 0.05). Serum IgE level was found to be significantly higher (*p* = 0.039) among adult patients (1189.08 ± 394.4) pg/ml than among healthy controls (459.21 ± 74.91) pg/ml.

### AD Patients Exhibit Significantly Lower Microbial Diversity Than Healthy Controls

Metagenomic sequencing was performed for 23 AD patients (11 adult, 12 pediatric) and 31 adult control samples who yielded adequate quantity (≥0.2 ng/μl) of microbial DNA (Robin et al., 2016). From quality filtered reads of the shotgun data (Supplementary Table 3), intra-individual variation measured by Shannon diversity was found to be significantly different between adult AD patients and controls (*p* = 0.02) (Table 1), but similar (*p* > 0.05) between adult and pediatric patients (Table 2).

**Table 1:** Relative abundance of core taxa between adult patients and healthy controls.

**Phyla**	**Genera**	**Species**	**Mean relative abundance of cases (%) ± StdDev**	**Mean relative abundance of controls (%) ± StdDev**	**Wilcoxon's *p*-value**	**Remarks**
			**(Min-Max)**	**(Min-Max)**		
Actinobacteria			7.1 ± 17.1 (0–56.2)	32.3 ± 29.1 (0–96.1)	**0.013**	Significantly higher in healthy controls
	***Cutibacterium***		0.9 ± 2.8 (0–9.3)	11.1 ± 22.1 (0–93.6)	**0.021**	Significantly higher in healthy controls
		***C. acnes***	0.9 ± 2.8 (0–9.3)	10.8 ± 21.9 (0–92.4)	**0.022**	
	***Corynebacterium***		0.5 ± 1.1 (0–3.7)	4.9 ± 5.9 (0–17.81)	**0.015**	Significantly higher in healthy controls
		***C. lipophiloflavum***	0.03 ± 0.1 (0–0.3)	2.16 ± 4 (0–15.4)	**0.01**	
	***Brachybacterium***		0.007 ± 0.02 (0–0.8)	1.3 ± 2.04 (0–8.4)	**0.03**	Significantly higher in healthy controls
		***Brachybacterium unclassified***	0.007 ± 0.02 (0–0.08)	1.22 ± 1.88 (0–7.7)	**0.003**	
	***Micrococcus***		3.82 ± 8.12 (0–23.3)	9.16 ± 12.7 (0–42.4)	0.09	Not significantly different between cases and controls
		***M. luteus***	3.82 ± 8.1 (0–23.3)	9.2 ± 12.6 (0–42.4)	0.09	
Basidiomycota			0.01 ± 0.01 (0–0.05)	8.05 ± 11.86 (0–47.89)	**0.001**	Significantly higher in healthy controls
	***Malassezia***		0.004 ± 0.02 (0–0.05)	8.9 ± 12.9 (0–48.9)	**0.001**	
		***M. globosa***	0.005 ± 0.02 (0–0.05)	8.89 ± 12.9 (0–48.9)	**0.001**	
Firmicutes^*^			28.64 ± 36.6 (0–99.4)	22.28 ± 28.38 (0–93.3)	0.89	Not significantly different between adult AD patients and healthy controls
	***Staphylococcus***		28.64 ± 36.7 (0–99.4)	21.8 ± 28.5 (0–93.3)	0.72	
						
		***S. hominis***	0.2 ± 0.4 (0–1.4)	16.43 ± 24.5 (0–89.8)	**0.002**	Significantly higher in Controls
		***S. aureus^#^***	22.17 ± 31.2 (0–94.3)	0 ± 0	NA	Present only in adult AD patients
		***S. epidermidis***	2.49 ± 4.4 (0–27.4)	2.8 ± 5.2 (0–20.4)	0.55	Not significantly different between adult AD patients and healthy controls
Proteobacteria^*^			59.49 ± 37.2 (0.19–98.9)	32.74 ± 39.1 (0–99.8)	0.07	Not significantly different between adult AD patients and healthy controls
	***Acinetobacter***		1.36 ± 2.6 (0–7.9)	5.89 ± 19.9 (0–98.7)	0.36	
	***Pseudomonas***		35.19 ± 43.47 (0–98.5)	13.76 ± 27.62 (0–97.1)	0.39	
		***P. stutzeri***	9.03 ± 29.7 (0–98.5)	11.7 ± 27.8 (0–97.0)	**0.01**	Significantly higher in healthy controls
Alpha Diversity (Shannon Index)^#^			1.01 ± 0.76 (0–2.3)	1.50 ± 0.80 (0.08–2.94)	**0.014**	

# *Present only in adult AD patients*.

* *Not significantly different between adult AD patients and healthy controls at the phylum level*.

*Bold p values are significant (<0.05)*.

**Table 2:** Relative abundance of core taxa between adult patients and pediatric patients.

**Phyla**	**Genera**	**Species**	**Mean relative abundance of adult cases (%) ± StdDev**	**Mean relative abundance of pedriati cases (%) ± StdDev**	**Wilcoxon's *p*-value**	**Remarks**
			**(Min-Max)**	**(Min-Max)**		
Actinobacteria			7.12 ± 17.1 (0–56.2)	11.15 ± 15.2 (0–45.3)	0.54	Not significantly different between adult and pediatric AD patients
	***Micrococcus***		3.82 ± 8.1 (0–23.3)	5.18 ± 8.2 (0–25.4)	1.00	
		***M. luteus***	3.81 ± 8.13 (0–41.9)	5.18 ± 8.22 (0–25.4)	1.00	
Firmicutes			28.64 ± 36.7 (0–99.4)	65.49 ± 36.8 (0–100)	**0.03**	Significantly higher in pediatric patients
	***Staphylococcus***		28.64 ± 36.7 (0–99.4)	63.68 ± 37.7 (0–100)	**0.04**	
		***S. aureus***	22.17 ± 31.2 (0–94.34)	32.86 ± 41.3 (0–98)	0.73	Not significantly different between adult and pediatric AD patients
		***S. epidermidis***	2.49 ± 4.37 (0–11.39)	16.37 ± 25.5 (0–63.5)	0.18	
Proteobacteria			59.5 ± 37.2 (0.2–100)	21.2+33.7 (0–100)	**0.02**	Significantly higher in adult patients
	***Pseudomonas***		35.19+43.5 (0–98.5)	12.90+35.7 (0–69.5)	0.08	Not significantly different between adult and paediatric AD patients
Alpha diversity (Shannon index)			1.01 ± 0.76 (0–2.3)	1.12+0.70 (0.1–2.30)	0.4	

*Bold p values are significant (<0.05)*.

**Figure 1:**
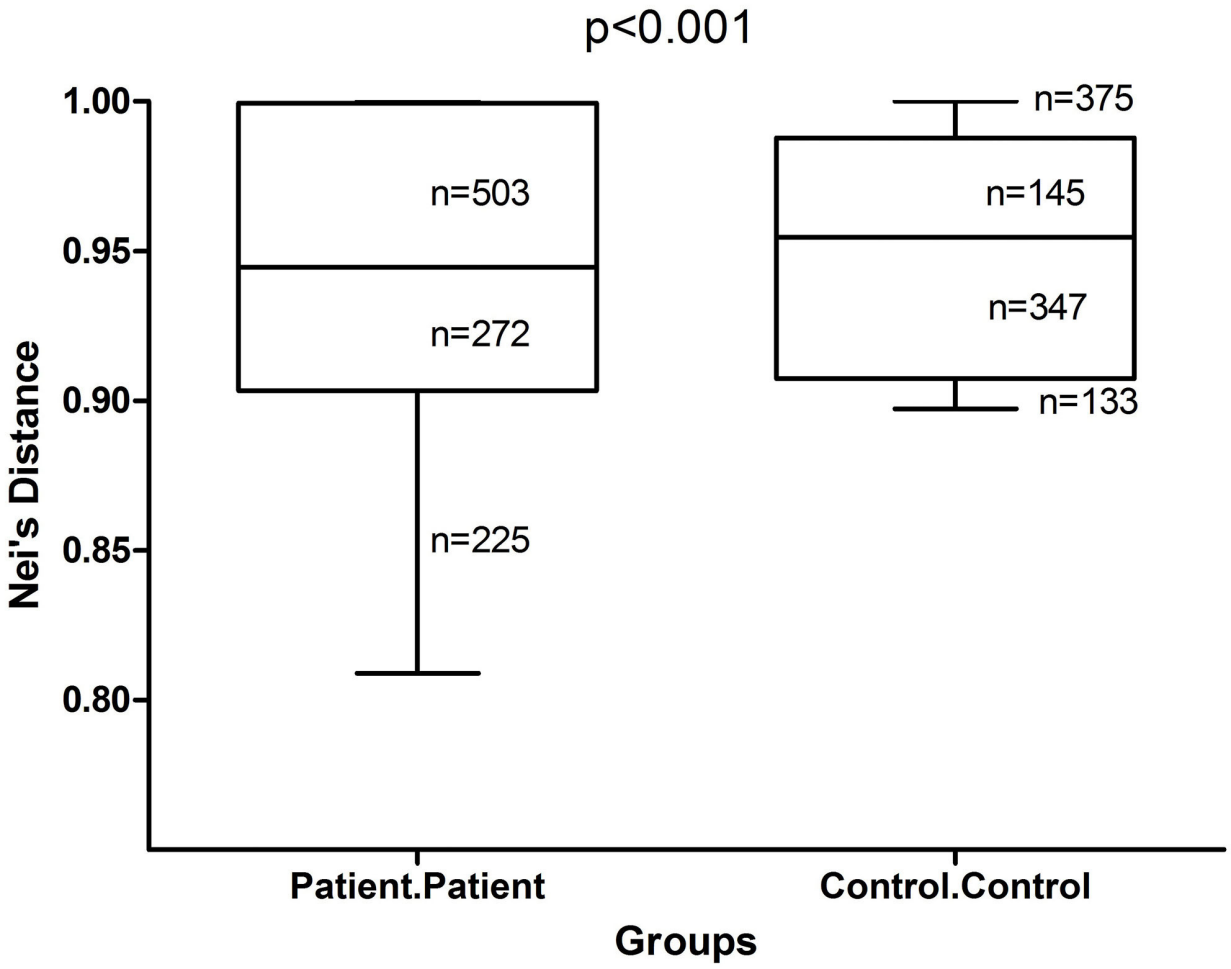
Box and whisker plots of Nei's distance in adult AD patients and healthy controls. Inter-individual variability among adult AD patients and healthy controls were measured by Nei's distance for 1,000 pairs of randomly sampled individuals from each group. The difference in the distribution of these pair-wise distances was tested for significance by Kolmogorov-Smirnov test. The Nei's distance was found to be significantly higher (*p* < 0.001) among healthy controls than among patients.

Inter-individual difference measured by Nei's distance, was significantly higher (*p* < 0.001) among healthy controls than among adult AD patients (Figure 1), consistent with findings of previous studies (Kong et al., 2012; Chng et al., 2016) that showed lower microbial diversity, enrichment of specific taxa with replacement of commensal taxa during the establishment of AD.

### *S. aureus* Predominates on AD Skin

Considerable reduction in number of taxa was noted in adult patients (*n*) compared to healthy controls (*m*) at the genus (*n* = 26; *m* = 77) and species levels (*n* = 46; *m* = 127). Among adult patients, three genera comprised more than 75% of the total relative abundance: *Pseudomonas* (35.19%), *Staphylococcus* (28.64%), and *Enhydrobacter* (13.40%). The predominant species were: *Pseudomonas stutzeri* (9.03%), *Staphylococcus aureus* (22.17%), and *Enhydrobacter aerosaccus* (13.40%). Among the healthy controls, seven genera were predominantly identified: *Staphylococcus* (21.83%), *Pseudomonas* (13.76%), *Propionibacterium* (11.14%), *Micrococcus* (9.16%), *Malassezia* (8.89%), *Acinetobacter* (5.88%), and *Corynebacterium* (4.93%) (Supplementary Table 4A). The predominant species were: *Staphylococcus hominis* (16.43%), *Pseudomonas stutzeri* (11.65%), *Cutibacterium acnes* (10.83%), *Micrococcus luteus* (9.16%), *Malassezia globosa* (8.89%), *Acinetobacter lwoffii* (5.05%), and *Corynebacterium lipophiloflavum* (2.15%) (Supplementary Table 4B). The core microbiome (mean relative abundance ≥1% and present in at least 50% of individuals in at least one group) at both the genus and species levels (eight and 10 respectively) were included for further statistical analysis.

*Staphylococcus aureus* was found to be abundant among both adult (22.17%) and pediatric (32.86%) patients, but was absent in age and gender matched adult healthy controls (Table 1).

**Figure 2:**
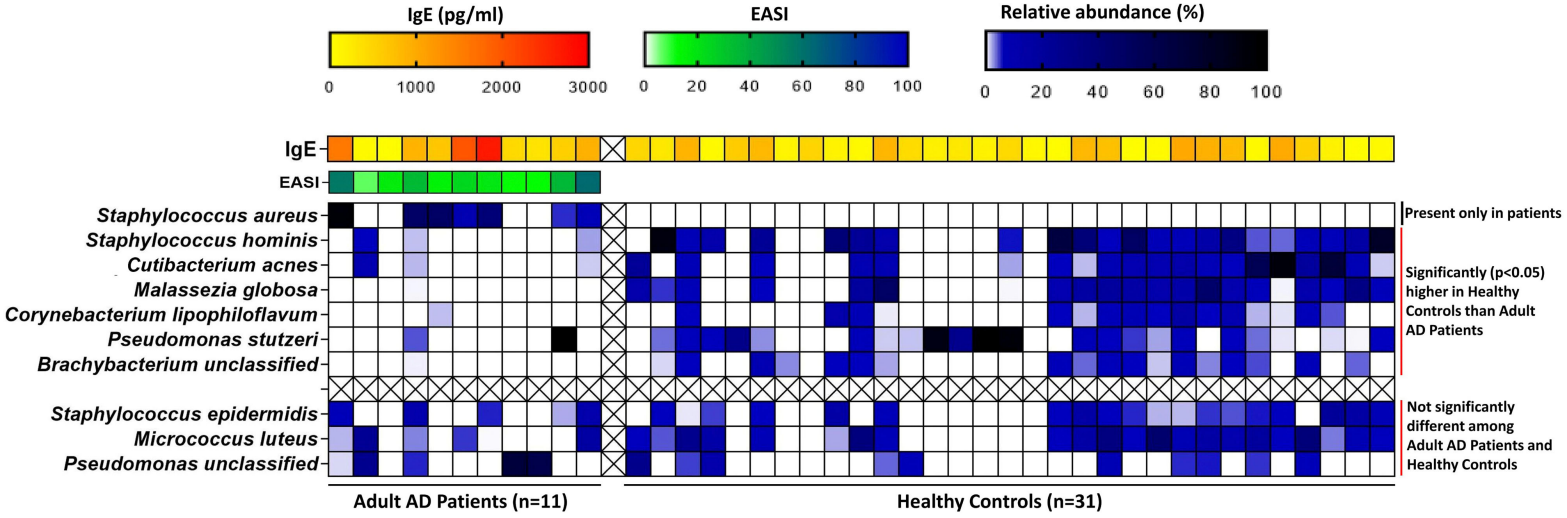
Heatmap of the relative abundances of core microbial species in adult AD patients and healthy controls along with other clinical factors like IgE (for both AD patients and controls) and disease severity index EASI (for AD patients only). The relative abundances of the core microbial species and serum IgE levels were compared between adult AD patients and Healthy controls by non-parametric Wilcoxon rank sum test. The blue-black gradient color band indicates the microbiome relative abundance (%). *Staphylococcus aureus* was absent in healthy controls. *Staphylococcus hominis, Cutibacterium acnes, Malassezia globosa, Corynebacterium lipophiloflavum, Pseudomonas stutzeri*, and *Brachybacterium unclassified* were found to be significantly higher in healthy controls compared to adult patients. The yellow-red gradient color band indicates the IgE concentration (pg/ml). IgE levels were significantly (*p* = 0.03) higher in adult patients compared to healthy controls. The green-blue color band indicates the EASI (Eczema Area and Severity Index) score. The distribution of EASI scores among adult patients are shown here.

The core microbiome in healthy skin were mostly commensal, that included *Staphylococcus hominis* (Healthy: 16.43%, AD: 0.20%; *p* = 0.002), *Cutibacterium acnes* (Healthy: 10.83%, AD: 0.90%; *p* = 0.02), *Corynebacterium Lipophiloflavum* (Healthy: 2.16%, AD: 0.03%; *p* = 0.01), and the fungus *Malassezia globosa* (Healthy: 8.89%, AD: 0.005%; *p* = 0.001) (Figure 2 and Table 1).

The predominance of *S. aureus* among patients but *S. hominis* among healthy controls can be attributed to the fact that *S. hominis* specifically inhibits *S. aureus* on the skin by production of some antimicrobial peptides (AMPs) that act synergistically with LL-37 (Nakatsuji et al., 2017). It has been reported (Nowicka and Grywalska, 2018) that among patients, deficiency of AMP production, change in lipid composition and increase in pH levels in the *stratum corneum* collectively contributes to the increased susceptibility and colonization of *S. aureus*.

Comparison of predominant taxa among adult and pediatric patients showed that phylum Proteobacteria was significantly (*p* = 0.02) more abundant among adult (59.49%) than among pediatric patients (21.20%). In contrast, phylum Firmicutes was significantly (*p* = 0.03) more abundant among pediatric (65.49%) than among adult patients (28.64%) (Table 2 and Supplementary Tables 4A,B). However, the microbiome profiles of adult and pediatric patients were generally similar at lower taxonomic levels and were therefore pooled for host-microbiome association study.

### Culture-Based Validation of Findings on *Staphylococcus aureus*

We have attempted to validate our sequencing-based observations on *S. aureus* by classical bacterial culturing. A total of 60 isolates were identified from all AD patients (*n* = 34) of which 46 were Gram positive cocci (GPC). The sample wise distribution of the GPC isolates were as follows: (a) 15 patients had 2 GPCs each, (b) 10 patients had 1 GPC each and (c) two patients had 3 GPCs each. The remaining seven patients did not show any GPCs in their culture isolates (Supplementary Tables 1, 5). *Staphylococcus aureus* was identified in 22 (48%; 22/46) out of 46 GPC isolates by their Catalase and Coagulase activities. A total of 49 isolates (39 GPCs) were identified from all the 54 healthy control individuals. The sample wise distribution of the GPC isolates were as follows: (a) 33 controls had 1 GPC each and (b) three controls had 2 GPCs each. The rest of the 18 control individuals did not show any GPCs in their culture isolates (Supplementary Table 5). None of the 39 GPCs isolated from healthy controls showed both catalase and coagulase activities, indicating the complete absence of *S. aureus* in the isolates, in agreement with the microbiome sequencing data. Nonparametric Spearman's rank correlation between CFU/ml values and relative abundances of *Staphylococcus aureus* for adult patients was positive and significant (rho = 0.88, *p* < 0.0002), and also for pediatric patients (rho = 0.75, *p* = 0.01). Thus, the presence of *S. aureus* and its abundance in patients was confirmed by both culture based and sequencing results.

### *Staphylococcus aureus* Abundance Is Positively Correlated With Serum IgE Levels in AD Patients

Spearman's rank correlation between core microbiome and IgE levels revealed significant positive correlation with species *Staphylococcus aureus* (rho = 0.48, *p* = 0.02) of phylum Firmicutes, among AD patients. This result remained significant (rho = 0.8, *p* = 0.006) for adult AD patients but not for pediatric patients and healthy controls, when compared separately.

Correlation of the other clinical parameters with core microbiome showed positive correlation of *Staphylococcus epidermidis* with disease severity index among all the patients (rho = 0.4, *p* = 0.08) as well as among adult AD patients (rho = 0.82, *p* = 0.0018) separately.

### MIP-1α, but Not Any Other Th1/Th-2-type Cytokine, Is Associated With Severity

Expression of the proinflammatory cytokine MIP-1α was significantly associated with disease severity (rho = 0.62, *p* = 0.001) among adult patients, but not among pediatric patients. The levels of MIP-1α expression (Mean ± S.E.M.) in the adult (4.71 ± 1.71) pg/ml and pediatric (4.47 ± 2.54) pg/ml AD cases and adult healthy controls (2.75 ± 0.4) pg/ml are shown in Supplementary Figure 1. Although the levels of MIP-1α was higher in adult AD cases (*n* = 11) than matched healthy controls (*n* = 11), yet it was not significantly different between the two groups (P_Wilcoxon_ = 0.3). According to Sanchez-Perez et al. (2013) those AD cases with disease severity index (EASI score) > 20 are termed as severe AD. When we compared the MIP-1α level of only severe AD cases (*n* = 6) with matched healthy controls (*n* = 6) by non-parametric Wilcoxon sign ranked test, we observed significant difference between the two groups (Mean ± S.E.M. for severe AD cases: 7.30 ± 2.82, matched controls: 2.06 ± 0.2, P_Wilcoxon_ = 0.03). MIP-1α has been previously shown to be a key factor associated with inflammation in AD (Menten et al., 2002).

### Microbial Pathways Enriched in AD Patients Are Associated With Skin Barrier Function and Ammonia Production

**Figure 3:**
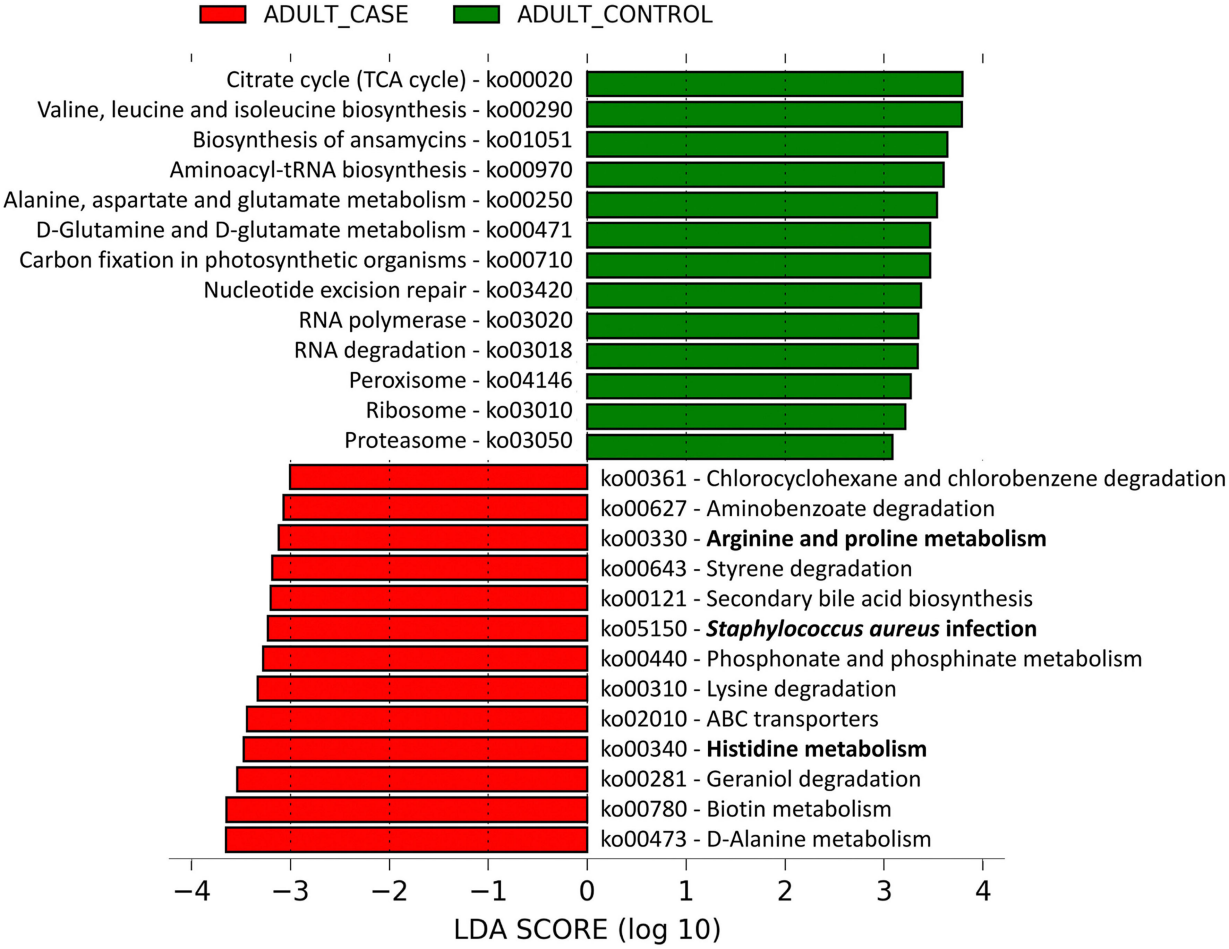
Linear discriminant analysis identified differentially abundant microbial pathways between adult AD patients and healthy controls. The microbial gene families identified from the shotgun metagenomic data were mapped to 182 microbial pathways by HUMAnN2.0. The relative abundances (Copies per Million) of these microbial pathways were compared between the adult AD patients and healthy controls by Linear Discriminant Analysis (LDA). Among them, 13 pathways were significantly enriched in adult AD patients (red colored) and another 13 were over-represented in the healthy controls (green colored). The pathways are denoted by unique ko (KEGG Ortholog) identifiers and the bold letters highlight those pathways that are associated with inflammation and dry skin.

Of the 182 microbial pathways identified at the community level from the metagenomic sequence data, linear discriminant analysis (LDA) showed 13 pathways to be over-represented in patients and 13 pathways to be over-represented in control individuals (Supplementary Tables 6A, B). On the AD lesional skin, significantly higher abundance of Arginine and Proline metabolism, Histidine metabolism and *Staphylococcus aureus* infection pathways were observed (Figure 3). These pathways were predominantly identified to be harbored by MRSA strains as follows: (a) *Staphylococcus aureus* infection pathway in MRSA strains COL, JH1, JH9, MW2, Mu3, N315, (b) Arginine and Proline, Histidine metabolism pathways in MRSA strains MRSA252, Mu3, N315.

The Arginine and Proline, Histidine metabolism pathways are known to produce ammonia which may increase the skin pH hindering the antimicrobial peptide activity on the skin surface. Arginine and histidine are also known to be major constituents of the skin structural protein FLG that maintains skin barrier integrity (Candi et al., 2005).

### *FLG* Null Mutations Are Less Frequent in the Indian AD Patients

We have identified 26 *FLG* single nucleotide variations (SNVs) to be commonly present among both AD patients (*n* = 23) and controls (*n* = 31) with metagenomic sequencing data. Of these SNVs, 20 (19 polymorphic) were missense, five were synonymous and one was identified as stop-gain null (W1064X) mutation.

*In silico* prediction of these single nucleotide polymorphisms (SNPs) using Variant Effect Predictor (VEP) of Ensembl (Mclaren et al., 2016) detected mutant alleles of 5 SNPs as “potentially damaging,” viz., rs41267154 (G>T), rs11588170 (G>A), rs11204978 (C>A), rs12407807 (G>A), and rs12405241 (C>T), in both as heterozygote and homozygote forms.

Only two *FLG* LoFs (2282del4 and W1064X) were identified among patients, of which, one was also present among controls. The percentage of AD cases and controls harboring at least one *FLG* LoF was similar among our study (13%, 3%) and the Europeans (23–45%, 7–8%) (Sandilands et al., 2007; Greisenegger et al., 2010) and other Asians (31%, 3%) (Nomura et al., 2007; Zhang et al., 2011; Park et al., 2015) (Table 3). However, the percentage of AD cases and controls carrying at least one AD associated *FLG* LoF was significantly lower (*p* < 0.005) in our study (8.6%, 0%) than those reported in Europeans (27%, 2.6%). We have also checked the whole genome sequence data on 533 randomly sampled Indians from the GenomeAsia100K (Wall et al., 2019) and observed the presence of only 2282del4 mutation (3/533; 0.6%) from among the six associated LoFs of *FLG* in them (Supplementary Table 7).

**Table 3:** Percentage of AD patients and Healthy controls with *FLG* LoFs in USA, Europe, and Asia.

**Global studies on AD**	**Study population**	**Patients carrying at least one *FLG* LoF (%)**	**Controls carrying at least one *FLG* LoF (%)**
Polcari et al. (2014)	United States (African American)	22.2% (4/18)	5.8% (1/17)
Greisenegger et al. (2010)	Austria and Germany	22.9% (106/462)	7.7% (31/402)
Sandilands et al. (2007)	Ireland	45.2% (85/188)	7.6% (56/736)
Zhang et al. (2011)	Han Chinese	31.4% (82/261)	0 (0/92)
Park et al. (2015)	Korean	16.04% (13/81)	NA
Nomura et al. (2007)	Japan	20.5% (21/102)	0 (0/156)
Our study	India	13% (3/23)	3.2% (1/31)

### Potentially Damaging Allele Dosages in *FLG* SNPs Are Negatively Correlated With *S. aureus* in AD Patients

Among the AD patients (*n* = 23) Spearman's rank correlation coefficient between the core microbiome abundance and summed damaging allele dosage of the 5 damaging SNPs revealed that phylum_Firmicutes up to species_*S. aureus* was significantly negatively correlated (rho < −0.47, *p* < 0.004) with the variant allele dosage (Supplementary Figure 2). Phylum_Proteobacteria up to Order_*Pseudomonadales* was also found to be significantly positively correlated (rho > 0.47, *p* < 0.02) with the variant allele dosage (Supplementary Figure 3). No significant correlation was observed between the potentially damaging allele dosage and core microbiome abundance among healthy controls.

### Microbial Pathways Involved in Impairing AMP Activity Thus Enhancing Skin Barrier Permeability Are Predominant in AD Patients Without Potentially Damaging Alleles in *FLG* SNPs

Among the 23 AD patients, those homozygous for the variant allele (*n* = 6) at all of the five potentially damaging *FLG* SNPs were compared with those homozygous (*n* = 4) for the reference allele at all these five loci for differences in abundance of core microbiome constituents as well as microbial pathways. This was done to understand the complexity of host-microbiome associations. *S. aureus* was significantly more abundant among homozygotes for the reference alleles than variant alleles [*S. aureus* abundance in; (a) Reference Allele Homozygotes: 53.6%, (b) Mutant allele Homozygotes: 2.2%. *p* = 0.01], supporting its negative correlation with the variant allele dosage reported above.

**Figure 4:**
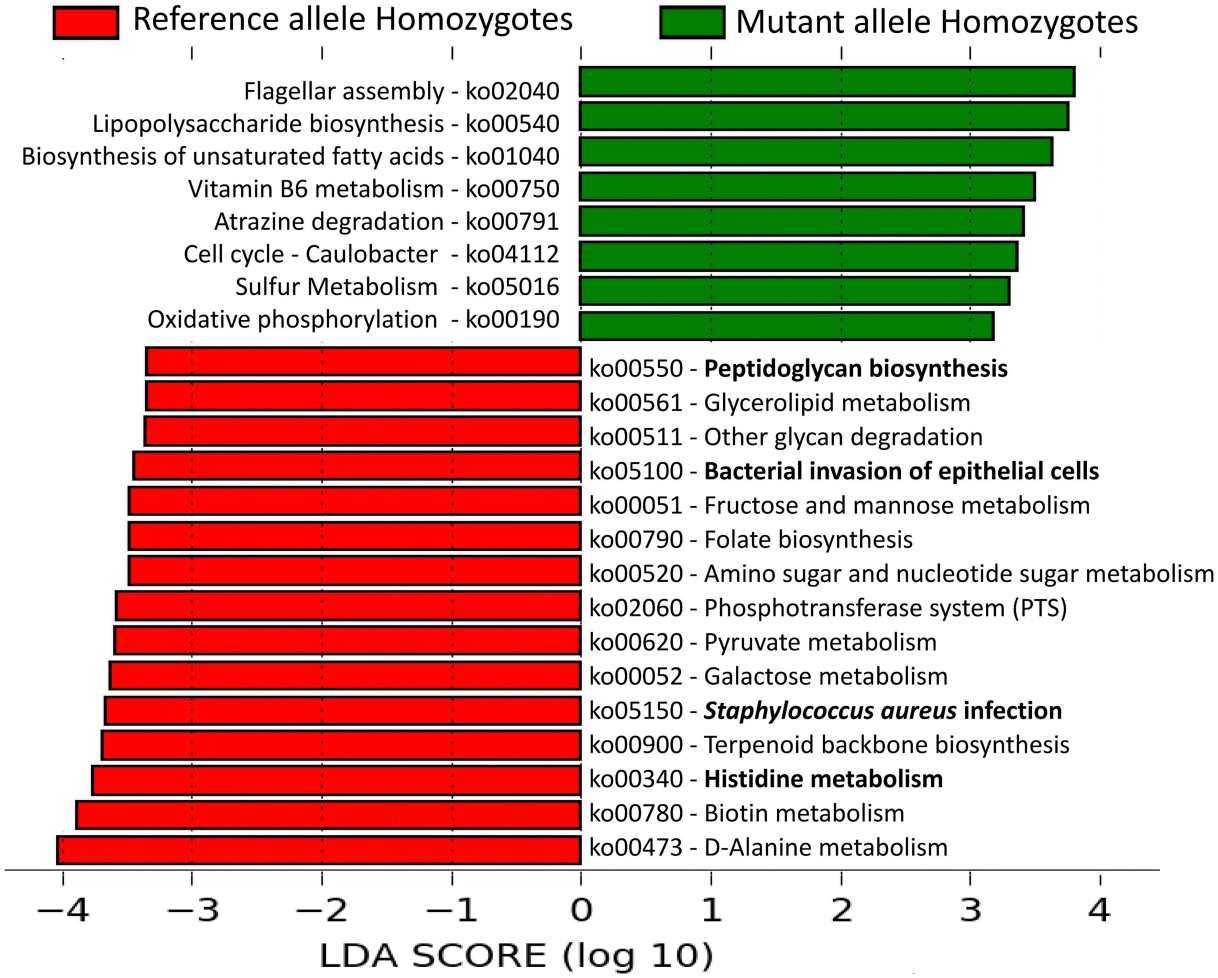
Linear Discriminant Analysis identified differentially abundant microbial pathways in AD patients with or without the potentially damaging *FLG* SNPs. The relative abundances (copies per million) of the microbial pathways were compared between AD patients that are reference allele homozygotes and mutant allele homozygotes for all the 5 “potentially damaging” missense SNPs in *FLG* gene. Among them, 15 pathways were significantly enriched in the reference allele homozygous patients whereas eight other pathways were over-represented in the mutant allele homozygous patients. The pathways are denoted by unique ko (KEGG Ortholog) identifiers and the bold letters highlight those pathways that are associated with inflammation and altered skin barrier permeability.

Microbial pathways enriched in the lesional microbiome of AD patients, viz., *Staphylococcus aureu*s infection and Histidine metabolism pathways, were enriched in the reference allele homozygote patients. Pathways, viz., bacterial invasion of epithelial cells and peptidoglycan biosynthesis, were also significantly enriched among these patients (Figure 4). All these pathways were unambiguously mapped to MRSA strains of *S. aureus*. These results provide strong evidence of significant association of *S. aureus* and its pathways in promoting impaired antimicrobial peptide activity and enhanced skin barrier permeability in the human host.

## Discussion

The results of this study, provided new insights on similarities and differences in skin microbiome composition between adult and pediatric AD patients. Although our results are similar to some earlier 16S studies (Kong et al., 2012; Shi et al., 2016), notable differences were found with previously reported results of the whole metagenome shotgun sequencing study of non-flare skin in AD patients (Chng et al., 2016). In the published study, the use of emollients that is known to alter microbiome profile (Glatz et al., 2018) was perhaps not restricted before collection of skin samples.

We found that *Staphylococcus aureus* (adult AD: 22.17%, pediatric AD: 32.86%, controls: 0%) was exclusively present on AD lesional skin of both adult and pediatric patients only. Capone et al. (2011) and Grice et al. (2009) have already shown that infant skin is dominated by Firmicutes (*Staphylococcus*) compared to adult skin due to the differences in the biophysical properties of the *stratum corneum* between the two age groups (Grice et al., 2009; Capone et al., 2011). Among healthy individuals, *Staphylococcus hominis* was highly abundant (adult AD: 0.25%, pediatric AD: 6.6%, controls: 16.5%). Antimicrobial peptides produced by *S. hominis* are known to inhibit growth or kill *S. aureus* by synergistic interaction with human LL37 (Nakatsuji et al., 2017). During AD, endogenous AMPs and cathelicidins are expressed at lower levels and this deficiency in AMP production may promote colonization of *S. aureus* (Nakatsuji et al., 2017). Recent studies have also shown that *S. epidermidis* may specifically limit the growth of *S. aureus* (Byrd et al., 2017). However, we have not observed any significant difference of relative abundance in *S. epidermidis* between healthy controls (2.8%) and AD patients (2.5%). The control skin also showed higher abundance of *Cutibacterium acnes* and *Malassezia globosa* both of which are known to be associated with high sebum concentration in the skin (Ro and Dawson, 2005; Mukherjee et al., 2016). These microbes degrade sebum to free fatty acids and consume them for carrying out their metabolic activities. Similar observation was found by Fyhrquist et al. (2019) where predominant bacteria in the AD and healthy skin were *S. aureus* and *Cutibacterium acnes* respectively (Fyhrquist et al., 2019). Pediatric patients showed a higher abundance of Firmicutes whereas Proteobacteria were more abundant among adult patients. A significant reduction in microbial diversity was observed on AD skin compared with normal skin (alpha diversity: Shannon index *p* = 0.02).

The microbial pathways significantly over-represented in AD lesional skin microbiome were Arginine and Proline metabolism, Histidine metabolism, *Staphylococcus aureus* infection, Lysine degradation and other xenobiotics (aminobenzoate, chlorocyclohexane, and chlorobenzene) degradation. The *Staphylococcus aureus* infection pathway is related to the functions like resistance to the antimicrobial peptides, inducing host inflammation and prevention of the membrane attack complex formation by the host against the microbes. This observation was concordant with Byrd et al. (2017) where whole genome sequencing of the *S. aureus* isolates identified from AD skin showed enrichment of *Staphylococcus aureus* infection pathway (Byrd et al., 2017). Over-representation of Arginine and Proline metabolism pathway in AD skin microbiome has been reported earlier (Chng et al., 2016). This, as well as the Histidine metabolism pathway, produces ammonia as a by-product that increases the skin pH thus making the skin vulnerable to opportunistic bacteria by deactivating the anti-microbial peptides (Cork and Danby, 2009; Chng et al., 2016). These pathways were involved in degradation of hygroscopic amino acids arginine and histidine derived from Filaggrin monomers. This may results in skin barrier disruption, trans-epidermal water loss (TEWL) and skin dryness (Hug et al., 1999; Cork and Danby, 2009), conditions that are precursors to AD. Species resolved pathways showed that Arginine and Proline metabolism, Histidine metabolism pathways were predominantly present in *S. aureus* among AD patients. Further strain level analysis identified MRSA strains (COL, JH1, JH9, MW2, Mu3, N315) to be present in AD skin microbiome with MW2 and N315 being the predominant strains that differentiate AD from healthy skin. This is similar to Byrd et al. (2017) where severe AD patients harbored MRSA strains (Byrd et al., 2017).

The structural protein Filaggrin, encoded by *FLG* on 1q21 in the gene-dense Epidermal Differentiation Complex, is involved in the maintenance of skin barrier function. During the formation of the *stratum corneum* the profilaggrin (precursor of Filaggrin protein) is dephosphorylated and proteolytically cleaved to form multiple copies of the functional Filaggrin peptide units. This helps in skin cornification by creating a strong barrier to retain in water and keep out allergens or bacterial toxins (O'Regan et al., 2008). Loss of function mutations (LoFs) in *FLG* impairs function of Filaggrin promoting disruption of skin barrier and penetration of allergens and irritants (Brown and Irwin Mclean, 2012). Six such LoFs had been found to be associated with atopic dermatitis in worldwide studies (R501X, 2282del4 in Europe, 3321delA, S2554X in Asia and S3316X, R826X in Africa) (Palmer et al., 2007; Sandilands et al., 2007; Enomoto et al., 2008; Winge et al., 2011; Margolis et al., 2018). However, except for 2282del4 found in two AD patients, none of the other five LoF mutations have been found in our patients or healthy controls. We have also checked the whole genome sequence data on 533 randomly sampled Indians from the GenomeAsia100K (Wall et al., 2019) and observed the presence of only 2282del4 mutation (3/533; 0.6%) from among the six associated LoFs of *FLG* in them. Thus, Filaggrin LoFs associated with AD in other global populations do not seem to play a major role in AD susceptibility among Indians. However, among the missense SNPs identified in *FLG* gene, five SNPs were found to harbor potentially damaging alleles which may alter the normal function of Filaggrin protein. Individuals with higher mutant allele dosages of the five potentially damaging missense SNPs had a higher abundance of phylum_Proteobacteria up to order_*Pseudomondales* (rho > 0.47, *p* < 0.02) and a lower abundance of phylum_Firmicutes up to species_*S. aureus* (rho < −0.47, *p* < 0.004) among AD patients.

Microbial pathways of *Staphylococcus aureus* related to interaction with host epithelial cells (Bacterial invasion of epithelial cells pathway) and pathways such as *Staphylococcus aureus* infection, Peptidoglycan biosynthesis and Histidine metabolism provided the maximal discrimination between patients with and without potentially damaging alleles by linear discriminant analysis. Peptidoglycan (PGN) predominate the cell wall of *S. aureus* and activate inflammatory response in keratinocytes via toll-like receptor 2. Previous findings indicate that cell wall products and toxins of staphylococci (such as peptidoglycans) modulate humoral immunity and may be responsible for allergic skin reactions in AD patients (Neuber and Konig, 1992). Functional studies have shown that *S. aureus* can express molecules such as α-Toxin which can mediate cytotoxic and immunological effects on host cells and cause direct cellular damage to keratinocytes (Song et al., 1996; Kobayashi and Nagao, 2019) leading to impaired lesional skin like conditions. A recent study (Zeeuwen et al., 2017) has shown that Gram positive anaerobic cocci (GPAC) mainly *Finegoldia, Anaerococcus*, and *Peptoniphilus* were significantly associated with the FLG+/+ skin of the Ichthyosis vulgaris (IV) patients. Using *in vitro* assays, this study also showed that FLG deficient skin was depleted for the bacterial taxa that utilizehistidine as a nutrient source due to loss of histidine rich FLG protein (Zeeuwen et al., 2017). Because of lack of *FLG* null mutations in our samples, we characterized the missense SNPs detected in the AD cases, and identified 5 SNPs to be carrying “potentially damaging” alleles by *in silico* prediction tools (VEP). We hypothesized that the damaging alleles adversely impacted on the stability of the FLG protein. Among the 23 AD patients, homozygotes for the reference allele at all the 5 SNP loci (*n* = 4) exhibited higher abundance of *Staphylococcus aureus* with gene families enriched for pathways related to histidine metabolism, *S. aureus* infection, peptidoglycan biosynthesis and bacterial invasion to epithelial cells compared to those patients who were homozygous for the variant (damaging) alleles. Based on this host-microbiome association, we have proposed the involvement of *S. aureus* and its pathways in influencing altered skin barrier function in the human host that leads to inflammation. Since the probability of observing a specific combination of genotypes at 5 loci is small and our overall sample size was also limited, we only observed four and six individuals in the two groups with specific 5-loci genotypic combinations. Hence, we also performed a correlation analysis between damaging allele dosage and abundance of *S. aureus* in all the 23 AD patients and found that among AD patients with a higher number of *FLG* damaging alleles, the abundance of *S. aureus* was lower. Our results are consistent with those of Zeeuwen et al. (2017) study that showed reduction of bacterial taxa that use histidine as a nutrient source among individuals with *FLG* null mutations.

**Figure 5:**
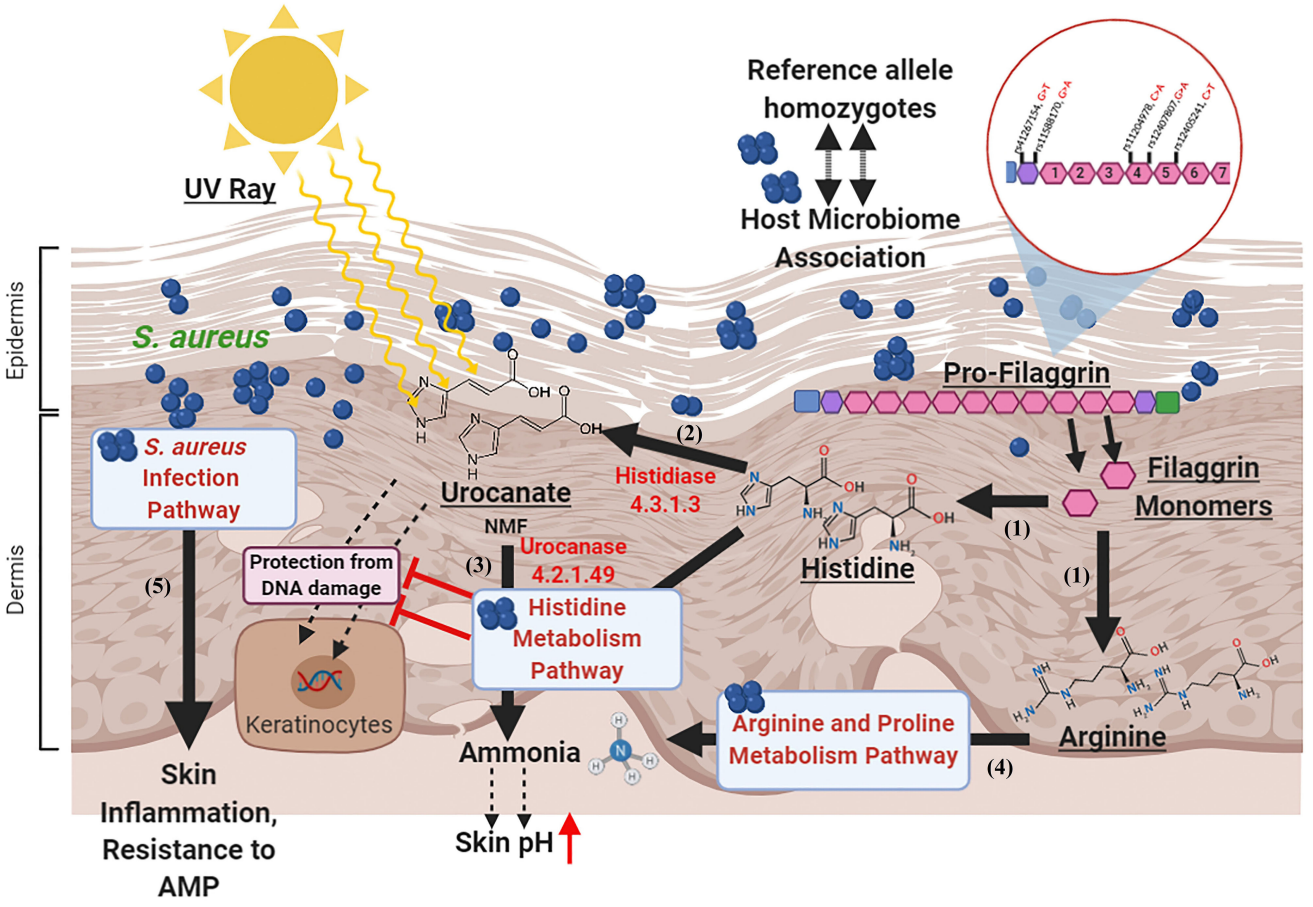
Proposed model for host microbiome association in atopic dermatitis. (1) Filaggrin monomers produce Histidine and Arginine (natural moisturizing factors) molecules in the epidermal and dermal layers upon degradation. (2) The Histidine is converted to Urocanate by Histidase enzyme in the skin which protects skin cells from DNA damage by absorbing UV rays. (3) In the AD patients with reference allele homozygous genotypes for all the 5 “potentially damaging” missense SNPs in *FLG* gene, the enzyme Urocanase present in the Staphylococcal Histidine metabolism pathway may help in degradation of Urocanate to produce ammonia that increases skin pH and impairs AMP activity. (4) In these individuals, the Arginine in the *stratum corneum* may be degraded by the enzymes in the Arginine and Proline metabolism pathway. (5) The *S. aureus* infection pathway may impair AMP activity and augment skin inflammation. Created with BioRender.com.

In the histidine metabolism pathway histidase (hutH) and urocanase (hutU) are the two enzymes that are involved in initial breakdown of the amino acid histidine (Bender, 2012). The enzyme histidase converts histidine to urocanate which has UV absorbing properties and can act as NMF (Barresi et al., 2011). Urocanate is subsequently converted to imidazolone propionate by the enzyme urocanase. Like histidase, this enzyme is widely distributed within the bacterial domain, but absent in most eukaryotes. It has been reported that animal epidermis contains histidase but no urocanase, resulting in accumulation of urocanate in skin (Zenisek et al., 1955; Tabachnick, 1957; Bender, 2012). Removal of this compound from skin by bacterial enzyme urocanase can negate its protective advantage. This supports our finding that alterations in the Histidine metabolism pathway is a major distinguishing feature between (a) AD cases and controls and (b) AD cases with or without the potentially damaging alleles of 5 SNPs (Figure 5).

The pathways (Bacterial invasion of epithelial cells, *Staphylococcus aureus* infection, Peptidoglycan biosynthesis and Histidine metabolism) were predominantly present in *S. aureus* and were highly abundant in those patients who are not carrying the potentially damaging alleles of the 5 SNPs. This suggests that although the AD patients who are reference allele homozygotes for the five potentially damaging SNPs retain their normal Filaggrin function but their skin integrity and homeostasis can be influenced by the dysbiotic microbiome and its altered biosynthetic pathways. These observations clearly indicate association between host genetic factors and microbiome profiles.

Our results indicate novel modes of host-microbiome association in India, possibly resulting from an evolutionary history that is distinct from Caucasian populations and perhaps also by unique environmental exposures. Among AD patients, microbiome profiles and their distinct microbial pathways were significantly altered in the presence or absence of five potentially damaging SNPs of *FLG* indicating a possible role of alteration of skin barrier permeability and the overall dysbiosis on the skin surface. Our study has also provided important insights on the involvement of lesional microbiome in the development of AD that utilize specific metabolic pathways of *S. aureus* which are directly linked to the elevation of skin pH and promoting inflammation.

## Data Availability Statement

The datasets generated for this study can be found in the EBI-ENA accession PRJEB40358.

## Ethics Statement

The studies involving human participants were reviewed and approved by The Institutional Ethics Committees of National Institute of Biomedical Genomics, Kalyani, India and Medical College and Hospital, Kolkata, India. Written informed consent to participate in this study was provided by the participants' legal guardian/next of kin.

## Author Contributions

PM, SM, and RM conceived the work. DB and NS recruited the study participants and performed clinical assessments. SN and SM collected the biospecimens and assisted with massively parallel sequencing. SN processed the biospecimens and carried out other wet-lab experiments. PM, SM, NK, and SN performed analyses of data. PM, SM, RM, NK, and SN wrote the manuscript. All authors have read and approved the manuscript. All authors contributed to the article and approved the submitted version.

## Conflict of Interest

RM is employed by Unilever R&D. The remaining authors declare that the research was conducted in the absence of any commercial or financial relationships that could be construed as a potential conflict of interest.
