# Supplementary material for: Dysbiotic Lesional Microbiome With Filaggrin Missense Variants Associate With Atopic Dermatitis in India
Source: Front Cell Infect Microbiol. 2020 Nov 17;10:570423. doi: 10.3389/fcimb.2020.570423 (PMC7705349; doi:10.3389/fcimb.2020.570423)
Supplement: Supplementary file 1 [file Data_Sheet_1.docx]

**Supplementary Material**

**Supplementary data**

**Skin Swab Sample Collection and Microbiome DNA isolation**

Lesional sites were chosen based on the severity of phenotypes such as xerosis, keratosis pilaris and visible flexural dermatitis as identified by an experienced dermatologist in the out-patients’ department of the hospital. The lesional swab was collected from that particular site of the AD patient where the phenotypes defining the AD were more severe than the rest of the body. This was done to successfully capture the lesional microbiome associated with AD in these patients. From each patient, lesional skin swab samples were collected using sterile cotton swabs in sterilized polypropylene tubes (HIMEDIA PW100). Before collection of samples, swabs were soaked in 1 ml of autoclaved 1X Phosphate buffer saline (pH 7.4) with TWEEN 20 (SIGMA) and gently rubbed by rotating at least two swab heads one at a time on the lesional sites of the AD Patients covering ~5 cm^2^ surface area ([Klymiuk et al., 2016](#_ENREF_4)). Site matched non-lesional skin swab samples were collected from the age and gender matched healthy control individuals from the same geographical region as the patients and using similar procedure as described above. The swab samples were then transported in cold condition to the laboratory in a sterile state and stored at -80^0^C until further processing. Microbiome DNA was isolated from skin swabs using QIAamp® BiOstic® Bacteremia DNA isolation kit following the manufacturer’s protocol. Swab samples were initially centrifuged for precipitating the microbial cells and then re-suspended in solution CB1 (Cell lysis buffer 1). Cell lysis was done both enzymatically and mechanically by vortexing with garnet beads (0.15 mm) provided with the kit. PCR inhibitors were removed by using inhibitor removal solution (IRS). After removal of inhibitors the solution was then passed through silica based spin filter column, washed and finally eluted in 50 µl of elution buffer (EB).Clinical information, including age of onset, Disease Severity (EASI) Index and Family History of Atopy and/or AD, was collected from the patients. Genomic DNA from the unclotted blood was isolated using QIAmp® Midi Kit following manufacturer’s protocol ([On et al., 2017](#_ENREF_5)).

**Shotgun Sequencing and Quality Filtering of the Raw reads**

Shotgun metagenomic sequencing was performed on a subset of AD patients and controls (Adult patients: 11, Paediatric patients: 12, Healthy controls: 31) with optimum DNA concentration (>0.2ng/ul) needed for generating the sequencing library ([Robin et al., 2016](#_ENREF_6)) using Nextera XT kit and sequenced by Illumina HiSeq2500 platform 2x250 bp paired end chemistry. A total number of 1109.72 M paired end raw reads were obtained with the average number of reads among Adult Patients (22.68+3.48M), Paediatric Patients (21.35+4.16 M) and Healthy controls (19.49+6.83M) being similar in nature. After removal of human reads 84.7% (17.36+ 6.3M) reads were retained of which 17.3% reads were removed due to quality filtering (Table S1).

**Cytokine Assay from Serum samples**

BioPlex assay was performed following the sandwich ELISA principle using magnetic beads containing the capture antibody and biotinylated detection antibody cross-linked with Phycoerythrin. The standard curves were generated with concentrations (pg/ml) for the standard antigens along X-axis and Median Fluorescence Intensity (MFI) along Y-axis. Unknown concentrations of the samples along X-axis were next estimated based on their MFI values along Y-axis following a five parameter equation ([Baud, 1993](#_ENREF_1)). Only those cytokines were included in the analysis whose readings from both the wells were within the detection limit. Only those samples were carried forward for further analyses whose inter individual variability in undiluted serum is greater than (>2 Standard Deviations) its intra individual variations ([Bhattacharyya et al., 2018](#_ENREF_2)). To identify differentially expressed cytokine profiles between (a) adult patients and adult controls and (b) adult and paediatric patients, non-parametric Wilcoxon rank-sum tests were performed.

**Statistical Analyses**

To compare the inter-individual variability across patients and controls, we randomly sampled with replacement 1000 pairs of individuals from within adult AD patients and healthy controls separately and computed Nei's distance between each pair. The difference in the distribution of these pair-wise distances was tested for significance by Kolmogorov-Smirnov test. To identify the change in microbiome abundance with disease severity (EASI), patients were classified as Moderate (n=5) (EASI within 7-20) and High (n=5) (EASI>20) groups ([Chopra et al., 2017](#_ENREF_3)) and Wilcoxon rank-sum test was performed at the genera and species levels.

**Table S1: Patient characteristics based on Williams Criteria**

| **Patient ID** | **Patient Group** | **Microbiome sequencing done** | **Williams Criteria for patient inclusion** | | | | | | **No. Of GPCs. Isolated per sample** |
| --- | --- | --- | --- | --- | --- | --- | --- | --- | --- |
|  |  |  | **Must** | **Any 3 Criteria to be satisfied** | | | | |  |
|  |  |  | **An Itchy Skin Condition** | **Disease onset below 2 yrs** | **Xerosis**  **(Dry skin)** | **Personal History of Other Atopic Disease** | **Keratosis pilaris** | **Visible Flexural Dermatitis** |  |
| AD003 | Adult | No | Yes | No | Yes | Yes | Yes | Yes | 3 |
| AD005 | Adult | Yes | Yes | Yes | Yes | Yes | Yes | Yes | 2 |
| AD006 | Adult | No | Yes | No | Yes | No | Yes | Yes | 2 |
| AD007 | Adult | Yes | Yes | Yes | Yes | Yes | Yes | Yes | 2 |
| AD009 | Adult | Yes | Yes | No | Yes | No | Yes | Yes | 1 |
| AD012 | Adult | Yes | Yes | No | Yes | No | Yes | Yes | 2 |
| AD013 | Adult | No | Yes | No | Yes | No | Yes | Yes | 2 |
| AD016 | Adult | No | Yes | No | Yes | No | Yes | Yes | 0 |
| AD017 | Adult | Yes | Yes | Yes | Yes | Yes | Yes | Yes | 2 |
| AD018 | Adult | No | Yes | No | Yes | No | Yes | Yes | 0 |
| AD022 | Adult | Yes | Yes | No | Yes | No | Yes | Yes | 1 |
| AD024 | Adult | No | Yes | No | Yes | No | Yes | Yes | 1 |
| AD025 | Adult | Yes | Yes | Yes | Yes | Yes | Yes | Yes | 2 |
| AD027 | Adult | Yes | Yes | No | Yes | No | Yes | Yes | 0 |
| AD028 | Adult | Yes | Yes | Yes | Yes | No | Yes | Yes | 1 |
| AD029 | Adult | Yes | Yes | Yes | Yes | Yes | Yes | Yes | 2 |
| AD030 | Adult | Yes | Yes | No | Yes | No | Yes | Yes | 2 |
| AD031 | Adult | No | Yes | No | Yes | Yes | Yes | Yes | 2 |
| AD001 | Paediatric | Yes | Yes | Yes | Yes | No | Yes | Yes | 2 |
| AD002 | Paediatric | Yes | Yes | Yes | Yes | No | Yes | Yes | 0 |
| AD004 | Paediatric | Yes | Yes | No | Yes | Yes | Yes | Yes | 0 |
| AD008 | Paediatric | Yes | Yes | No | Yes | No | Yes | Yes | 2 |
| AD010 | Paediatric | Yes | Yes | Yes | Yes | No | Yes | Yes | 1 |
| **Patient ID** | **Patient Group** | **Microbiome sequencing done** | **Williams Criteria for patient inclusion** | | | | | | **No. Of GPCs. Isolated per sample** |
|  |  |  | **Must** | **Any 3 Criteria to be satisfied** | | | | |  |
|  |  |  | **An Itchy Skin Condition** | **Disease onset below 2 yrs** | **Xerosis**  **(Dry skin)** | **Personal History of Other Atopic Disease** | **Keratosis pilaris** | **Visible Flexural Dermatitis** |  |
| AD011 | Paediatric | Yes | Yes | No | Yes | No | Yes | Yes | 2 |
| AD014 | Paediatric | No | Yes | No | Yes | No | Yes | Yes | 3 |
| AD015 | Paediatric | Yes | Yes | No | Yes | Yes | Yes | Yes | 2 |
| AD019 | Paediatric | Yes | Yes | No | Yes | No | Yes | Yes | 1 |
| AD020 | Paediatric | Yes | Yes | Yes | Yes | No | Yes | Yes | 1 |
| AD021 | Paediatric | No | Yes | Yes | Yes | No | Yes | Yes | 1 |
| AD023 | Paediatric | No | Yes | No | Yes | No | Yes | Yes | 0 |
| AD026 | Paediatric | Yes | Yes | No | Yes | Yes | Yes | Yes | 0 |
| AD032 | Paediatric | Yes | Yes | No | Yes | No | Yes | Yes | 1 |
| AD033 | Paediatric | Yes | Yes | No | Yes | Yes | Yes | Yes | 2 |
| AD034 | Paediatric | No | Yes | Yes | Yes | No | Yes | Yes | 1 |

**Table S2: Comparison of Microbiome composition between two sampling sites**

| **Taxa** | **AD Adult Patients** | | |  | **AD Paediatric Patients** | | |  | **Adult Healthy Control** | | |
| --- | --- | --- | --- | --- | --- | --- | --- | --- | --- | --- | --- |
|  | Antecubital fossa (n=5)  MRA(%) | Cervical Region  (n=6)  MRA(%) | Wilcoxon p value |  | Antecubital fossa (n=9)  MRA(%) | Cervical Region  (n=3)  MRA(%) | Wilcoxon p value |  | Antecubital fossa (n=16)  MRA(%) | Cervical Region  (n=15)  MRA(%) | Wilcoxon p value |
| p__Actinobacteria | 11.729 | 3.296 | 0.45 |  | 13.027 | 5.526 | 0.85 |  | 39.227 | 25.000 | 0.07 |
| p__Firmicutes | 29.809 | 27.674 | 0.93 |  | 65.185 | 66.411 | 0.85 |  | 27.045 | 17.207 | 0.15 |
| p__Proteobacteria | 49.960 | 67.435 | 0.71 |  | 19.398 | 26.641 | 1.00 |  | 19.649 | 46.707 | 0.29 |
| p__Basidiomycota | 0.000 | 0.009 | 0.47 |  | 0.012 | 0.000 | 0.70 |  | 10.013 | 7.698 | 0.13 |
| *g__Corynebacterium* | 0.739 | 0.298 | 0.60 |  | 0.004 | 3.064 | 0.05 |  | 6.135 | 3.649 | 0.15 |
| *g__Brachybacterium* | 0.000 | 0.013 | 0.47 |  | 1.542 | 0.000 | 0.23 |  | 1.919 | 0.649 | 0.06 |
| *g__Micrococcus* | 4.946 | 2.874 | 0.29 |  | 6.355 | 1.675 | 0.54 |  | 12.149 | 5.979 | 0.05 |
| *g__Propionibacterium* | 1.865 | 0.101 | 0.91 |  | 0.256 | 0.035 | 0.57 |  | 8.324 | 14.151 | 0.56 |
| *g__Staphylococcus* | 29.809 | 27.672 | 0.93 |  | 63.567 | 64.012 | 0.93 |  | 26.215 | 17.168 | 0.16 |
| *g__Acinetobacter* | 1.584 | 1.164 | 0.37 |  | 2.689 | 0.000 | 0.33 |  | 7.314 | 4.371 | 0.31 |
| *g__Pseudomonas* | 36.463 | 34.138 | 0.93 |  | 9.970 | 21.705 | 1.00 |  | 6.219 | 21.806 | 0.95 |
| *g__Malassezia* | 0.000 | 0.009 | 0.47 |  | 0.012 | 0.000 | 0.70 |  | 10.013 | 7.698 | 0.13 |
| *s__Corynebacterium_lipophiloflavum* | 0.000 | 0.053 | 0.47 |  | 0.000 | 0.000 | NA |  | 2.686 | 1.596 | 0.19 |
| *s__Brachybacterium_unclassified* | 0.000 | 0.013 | 0.47 |  | 1.494 | 0.000 | 0.23 |  | 1.799 | 0.614 | 0.06 |
| *s__Micrococcus_luteus* | 4.946 | 2.874 | 0.29 |  | 6.355 | 1.675 | 0.54 |  | 12.149 | 5.979 | 0.05 |
| *s__Propionibacterium_acnes* | 1.865 | 0.101 | 0.91 |  | 0.252 | 0.035 | 0.57 |  | 8.228 | 13.619 | 0.45 |
| *s__Staphylococcus_aureus* | 28.285 | 17.075 | 0.93 |  | 32.460 | 34.065 | 0.71 |  | 0.000 | 0.000 | NA |
| *s__Staphylococcus_epidermidis* | 0.995 | 3.741 | 0.69 |  | 14.630 | 21.589 | 0.45 |  | 1.670 | 3.932 | 0.49 |
| *s__Staphylococcus_hominis* | 0.283 | 0.130 | 0.91 |  | 7.516 | 6.758 | 1.00 |  | 21.138 | 11.415 | 0.13 |
| *s__Pseudomonas_stutzeri* | 0.000 | 16.565 | 0.22 |  | 1.379 | 0.000 | 0.48 |  | 3.154 | 20.732 | 0.83 |
| *s__Pseudomonas_unclassified* | 19.089 | 10.315 | 0.49 |  | 5.115 | 0.000 | 0.70 |  | 3.065 | 0.670 | 0.15 |
| *s__Malassezia_globosa* | 0.000 | 0.009 | 0.47 |  | 0.012 | 0.000 | 0.70 |  | 10.013 | 7.698 | 0.13 |

***MRA: Mean Relative Abundance**

**Table S3: Initial QA/QC of Microbiome Sequencing Reads**

| **Sample ID** | **Total no. of P-E Reads (in Millions)** | **Total no. of Human P-E reads** | | **P-E reads removed due to Short Length (<36 bp)** | | **P-E reads removed due to Lower QV (<15)** | | **Total P-E reads removed due to QA/QC** | | **Total no. of Microbial P-E reads (in Millions)** | |
| --- | --- | --- | --- | --- | --- | --- | --- | --- | --- | --- | --- |
|  |  | **in Millions** | **%** | **in Millions** | **%** | **in Millions** | **%** | **in Millions** | **%** | **in Millions** | **%** |
| AD005 | 23.89 | 9.32 | 39.01 | 0.90 | 3.76 | 0.69 | 2.87 | 1.58 | 6.63 | 12.98 | 54.36 |
| AD007 | 27.98 | 15.48 | 55.34 | 0.71 | 2.54 | 0.61 | 2.17 | 1.32 | 4.71 | 11.18 | 39.96 |
| AD009 | 17.98 | 7.56 | 42.07 | 0.41 | 2.29 | 0.53 | 2.96 | 0.94 | 5.25 | 9.47 | 52.68 |
| AD012 | 24.02 | 8.53 | 35.53 | 0.93 | 3.88 | 0.82 | 3.40 | 1.75 | 7.28 | 13.74 | 57.19 |
| AD017 | 18.29 | 7.09 | 38.78 | 0.47 | 2.56 | 0.63 | 3.44 | 1.10 | 6.00 | 10.10 | 55.22 |
| AD022 | 25.22 | 10.62 | 42.10 | 0.59 | 2.33 | 0.71 | 2.80 | 1.30 | 5.14 | 13.31 | 52.76 |
| AD025 | 27.65 | 0.26 | 0.94 | 4.73 | 17.10 | 1.69 | 6.12 | 6.42 | 23.22 | 20.97 | 75.84 |
| AD027 | 19.61 | 0.00 | 0.02 | 3.33 | 17.00 | 1.05 | 5.35 | 4.38 | 22.35 | 15.22 | 77.63 |
| AD028 | 21.47 | 0.00 | 0.01 | 3.68 | 17.16 | 1.14 | 5.30 | 4.82 | 22.46 | 16.65 | 77.53 |
| AD029 | 18.84 | 0.87 | 4.62 | 1.96 | 10.40 | 1.07 | 5.67 | 3.03 | 16.07 | 14.94 | 79.31 |
| AD030 | 24.52 | 7.86 | 32.06 | 1.16 | 4.75 | 0.91 | 3.72 | 2.08 | 8.47 | 14.58 | 59.47 |
| AD005A | 14.41 | 0.09 | 0.63 | 2.23 | 15.48 | 1.42 | 9.82 | 3.65 | 25.30 | 10.68 | 74.07 |
| AD005C | 19.24 | 0.24 | 1.25 | 3.01 | 15.63 | 1.03 | 5.35 | 4.03 | 20.97 | 14.96 | 77.78 |
| AD005D | 19.71 | 1.15 | 5.82 | 2.25 | 11.41 | 0.74 | 3.75 | 2.99 | 15.16 | 15.57 | 79.02 |
| AD007A | 15.70 | 0.02 | 0.15 | 2.43 | 15.48 | 1.50 | 9.58 | 3.93 | 25.06 | 11.74 | 74.79 |
| AD007C | 20.78 | 0.00 | 0.02 | 3.53 | 17.00 | 1.70 | 8.18 | 5.23 | 25.18 | 15.55 | 74.80 |
| AD007D | 20.61 | 0.32 | 1.57 | 2.61 | 12.66 | 0.82 | 3.96 | 3.42 | 16.61 | 16.86 | 81.81 |
| AD009B | 24.19 | 0.00 | 0.01 | 4.74 | 19.60 | 1.68 | 6.95 | 6.42 | 26.55 | 17.77 | 73.44 |
| AD009C | 25.38 | 0.01 | 0.02 | 4.21 | 16.58 | 1.50 | 5.91 | 5.71 | 22.49 | 19.67 | 77.49 |
| AD009D | 15.59 | 3.17 | 20.34 | 0.79 | 5.06 | 0.59 | 3.77 | 1.38 | 8.83 | 11.04 | 70.83 |
| AD012D | 17.52 | 0.75 | 4.26 | 2.14 | 12.22 | 0.59 | 3.37 | 2.73 | 15.59 | 14.05 | 80.15 |
| AD012E | 51.14 | 1.46 | 2.86 | 6.36 | 12.43 | 1.79 | 3.50 | 8.15 | 15.94 | 41.53 | 81.20 |
| AD017A | 12.78 | 0.01 | 0.08 | 1.69 | 13.23 | 0.98 | 7.70 | 2.67 | 20.92 | 10.09 | 79.00 |
| **Sample ID** | **Total no. of P-E Reads (in Millions)** | **Total no. of Human P-E reads** | | **P-E reads removed due to Short Length (<36 bp)** | | **P-E reads removed due to Lower QV (<15)** | | **Total P-E reads removed due to QA/QC** | | **Total no. of Microbial P-E reads (in Millions)** | |
|  |  | **in Millions** | **%** | **in Millions** | **%** | **in Millions** | **%** | **in Millions** | **%** | **in Millions** | **%** |
| AD017B | 19.25 | 0.01 | 0.08 | 3.76 | 19.55 | 10.33 | 53.66 | 4.77 | 24.80 | 14.46 | 75.12 |
| AD017C | 16.90 | 0.01 | 0.03 | 2.77 | 16.36 | 1.26 | 7.44 | 4.02 | 23.76 | 12.88 | 76.20 |
| AD018A | 16.22 | 0.00 | 0.02 | 2.74 | 16.90 | 1.13 | 6.94 | 3.87 | 23.84 | 12.35 | 76.14 |
| AD018B | 16.51 | 0.01 | 0.05 | 2.68 | 16.21 | 1.22 | 7.39 | 3.90 | 23.61 | 12.61 | 76.35 |
| AD022A | 14.07 | 0.00 | 0.02 | 2.13 | 15.15 | 0.65 | 4.63 | 2.78 | 19.79 | 11.28 | 80.19 |
| AD022B | 19.36 | 1.07 | 5.55 | 2.34 | 12.08 | 0.70 | 3.64 | 3.04 | 15.72 | 15.24 | 78.73 |
| AD022C | 19.98 | 6.32 | 31.62 | 1.04 | 5.23 | 0.58 | 2.91 | 1.62 | 8.13 | 12.03 | 60.25 |
| AD025A | 14.59 | 0.94 | 6.45 | 1.37 | 9.36 | 0.67 | 4.56 | 2.03 | 13.92 | 11.62 | 79.63 |
| AD025B | 17.77 | 0.94 | 5.27 | 2.08 | 11.70 | 0.56 | 3.15 | 2.64 | 14.84 | 14.19 | 79.89 |
| AD025C | 19.40 | 1.37 | 7.07 | 2.12 | 10.94 | 0.73 | 3.76 | 2.85 | 14.71 | 15.18 | 78.22 |
| AD025D | 17.91 | 0.76 | 4.24 | 2.21 | 12.34 | 0.70 | 3.89 | 2.91 | 16.22 | 14.25 | 79.53 |
| AD027A | 21.25 | 1.47 | 6.91 | 2.03 | 9.53 | 0.98 | 4.59 | 3.00 | 14.12 | 16.78 | 78.96 |
| AD027B | 19.30 | 0.25 | 1.29 | 2.52 | 13.08 | 0.76 | 3.93 | 3.28 | 17.01 | 15.77 | 81.70 |
| AD027D | 14.11 | 0.95 | 6.70 | 1.58 | 11.22 | 0.60 | 4.28 | 2.19 | 15.51 | 10.98 | 77.79 |
| AD028A | 29.46 | 7.15 | 24.28 | 1.79 | 6.09 | 0.93 | 3.16 | 2.73 | 9.25 | 19.58 | 66.47 |
| AD028B | 16.74 | 5.32 | 31.77 | 0.93 | 5.54 | 0.56 | 3.33 | 1.49 | 8.87 | 9.94 | 59.36 |
| AD028C | 18.84 | 3.87 | 20.55 | 1.45 | 7.69 | 0.62 | 3.27 | 2.06 | 10.96 | 12.90 | 68.50 |
| AD029A | 16.30 | 1.92 | 11.77 | 1.38 | 8.44 | 0.63 | 3.84 | 2.00 | 12.28 | 12.38 | 75.94 |
| AD030A | 19.08 | 2.05 | 10.75 | 2.08 | 10.90 | 0.61 | 3.18 | 2.69 | 14.08 | 14.34 | 75.17 |
| AD001 | 22.37 | 7.99 | 35.72 | 0.94 | 4.19 | 0.76 | 3.38 | 1.69 | 7.56 | 12.69 | 56.72 |
| AD002 | 22.57 | 9.04 | 40.08 | 0.55 | 2.45 | 0.67 | 2.95 | 1.22 | 5.40 | 12.30 | 54.52 |
| AD004 | 16.22 | 5.32 | 32.79 | 0.37 | 2.30 | 0.58 | 3.57 | 0.95 | 5.87 | 9.95 | 61.34 |
| AD008 | 17.47 | 6.33 | 36.25 | 0.38 | 2.20 | 0.59 | 3.40 | 0.98 | 5.60 | 10.16 | 58.15 |
| AD010 | 22.19 | 6.28 | 28.31 | 1.40 | 6.30 | 0.90 | 4.07 | 2.30 | 10.37 | 13.61 | 61.33 |
| **Sample ID** | **Total no. of P-E Reads (in Millions)** | **Total no. of Human P-E reads** | | **P-E reads removed due to Short Length (<36 bp)** | | **P-E reads removed due to Lower QV (<15)** | | **Total P-E reads removed due to QA/QC** | | **Total no. of Microbial P-E reads (in Millions)** | |
|  |  | **in Millions** | **%** | **in Millions** | **%** | **in Millions** | **%** | **in Millions** | **%** | **in Millions** | **%** |
| AD011 | 14.92 | 5.98 | 40.06 | 0.38 | 2.58 | 0.43 | 2.88 | 0.81 | 5.46 | 8.13 | 54.48 |
| AD015 | 20.92 | 7.19 | 34.37 | 1.41 | 6.74 | 1.03 | 4.92 | 2.44 | 11.66 | 11.29 | 53.97 |
| AD019 | 23.65 | 0.54 | 2.28 | 3.24 | 13.68 | 1.58 | 6.68 | 4.81 | 20.36 | 18.30 | 77.36 |
| AD020 | 23.91 | 0.02 | 0.09 | 3.35 | 14.03 | 1.12 | 4.66 | 4.47 | 18.69 | 19.42 | 81.22 |
| AD026 | 30.83 | 0.00 | 0.01 | 5.50 | 17.85 | 1.81 | 5.88 | 7.31 | 23.73 | 23.51 | 76.26 |
| AD032 | 19.86 | 6.47 | 32.57 | 0.44 | 2.21 | 0.58 | 2.94 | 1.02 | 5.14 | 12.37 | 62.29 |
| AD033 | 21.25 | 8.10 | 38.13 | 1.04 | 4.88 | 0.42 | 2.00 | 1.46 | 6.88 | 11.68 | 54.99 |
| **Average** | **20.55** | **3.19** | **15.23** | **2.09** | **10.15** | **1.09** | **5.45** | **3.01** | **14.71** | **14.35** | **70.06** |
| **Total** | **1109.72** | **172.52** | **NA** | **112.87** | **NA** | **58.84** | **NA** | **162.39** | **NA** | **774.81** | **NA** |

**Table S4A: Mean relative abundances of all genera identified in Adult AD patients, Paediatric AD patients and Healthy control individuals**

| **Genus** | **Adult patients  (Mean Relative Abundance %)** | **Paediatric patients  (Mean Relative Abundance %)** | **Healthy controls  (Mean Relative Abundance %)** |
| --- | --- | --- | --- |
| *Achromobacter* | 0.000 | 0.494 | 0.000 |
| *Acinetobacter* | 1.355 | 2.017 | 5.890 |
| *Actinomyces* | 0.000 | 0.149 | 0.020 |
| *Aerococcus* | 0.001 | 0.000 | 0.058 |
| *Agrobacterium* | 3.020 | 0.152 | 0.166 |
| *Ahjdlikevirus* | 0.000 | 0.000 | 0.039 |
| *Alcaligenes* | 0.000 | 0.294 | 0.000 |
| *Alishewanella* | 0.000 | 0.000 | 0.010 |
| *Alloprevotella* | 0.000 | 0.000 | 0.005 |
| *Alpharetrovirus* | 0.000 | 0.000 | 0.039 |
| *Aspergillaceae_unclassified* | 0.007 | 0.000 | 0.016 |
| *Asticcacaulis* | 0.000 | 0.008 | 0.031 |
| *Atopobium* | 0.000 | 0.086 | 0.003 |
| *Bacillus* | 0.000 | 0.000 | 0.032 |
| *Betapapillomavirus* | 0.000 | 0.000 | 0.091 |
| *Brachybacterium* | 0.007 | 1.157 | 1.305 |
| *Brevibacterium* | 1.800 | 1.604 | 0.295 |
| *Brevundimonas* | 0.472 | 2.116 | 5.262 |
| *Brucella* | 0.033 | 0.789 | 0.003 |
| *C2likevirus* | 0.000 | 0.018 | 0.000 |
| *Chroococcidiopsis* | 0.000 | 0.000 | 0.174 |
| *Comamonas* | 0.010 | 0.200 | 0.277 |
| *Corynebacterium* | 0.499 | 0.769 | 4.932 |
| *Debaryomyces* | 0.000 | 0.047 | 0.000 |
| *Deinococcus* | 1.020 | 0.532 | 0.351 |
| *Delftia* | 2.291 | 0.140 | 1.586 |
| *Dermabacteraceae_unclassified* | 0.000 | 0.243 | 0.466 |
| *Dermatophilaceae_unclassified* | 0.000 | 0.000 | 0.873 |
| *Dietzia* | 0.099 | 0.000 | 2.118 |
| *Enhydrobacter* | 13.406 | 0.000 | 1.775 |
| *Erythrobacteraceae_unclassified* | 0.000 | 0.000 | 0.149 |
| *Exiguobacterium* | 0.000 | 0.000 | 0.036 |
| *Facklamia* | 0.000 | 0.000 | 0.003 |
| *Finegoldia* | 0.000 | 0.234 | 0.000 |
| *Gammaretrovirus* | 0.000 | 0.000 | 0.002 |
| *Gardnerella* | 0.000 | 0.317 | 0.004 |
| *Gemella* | 0.000 | 0.000 | 0.008 |
| *Geodermatophilaceae_unclassified* | 0.000 | 0.000 | 0.001 |
| *Gordonia* | 0.000 | 0.000 | 0.014 |
| *Granulicatella* | 0.000 | 0.130 | 0.011 |
| **Genus** | **Adult patients  (Mean Relative Abundance %)** | **Paediatric patients  (Mean Relative Abundance %)** | **Healthy controls  (Mean Relative Abundance %)** |
| *Haemophilus* | 0.000 | 0.000 | 0.015 |
| *Halococcus* | 0.000 | 0.012 | 0.016 |
| *Halomonas* | 0.000 | 0.000 | 0.068 |
| *Janibacter* | 0.000 | 0.560 | 0.383 |
| *Klebsiella* | 0.000 | 0.000 | 0.017 |
| *Kocuria* | 0.000 | 0.082 | 0.016 |
| *Kytococcus* | 0.000 | 0.160 | 0.484 |
| *Lautropia* | 0.000 | 0.013 | 0.000 |
| *Malassezia* | 0.005 | 0.009 | 8.893 |
| *Marinococcus* | 0.000 | 0.000 | 0.079 |
| *Massilia* | 0.000 | 0.062 | 0.051 |
| *Micrococcus* | 3.816 | 5.185 | 9.163 |
| *Microcoleus* | 0.000 | 0.000 | 0.051 |
| *Natronococcus* | 0.000 | 0.000 | 0.037 |
| *Neisseria* | 0.000 | 1.060 | 0.078 |
| *Nocardioides* | 0.000 | 0.000 | 0.071 |
| *Oceanobacillus* | 0.000 | 0.000 | 0.009 |
| *Ochrobactrum* | 0.782 | 0.000 | 0.000 |
| *Pantoea* | 0.000 | 0.000 | 0.002 |
| *Papillomaviridae_noname* | 0.595 | 0.000 | 0.000 |
| *Paracoccus* | 0.000 | 0.253 | 0.383 |
| *Penicillium* | 0.000 | 1.485 | 1.378 |
| *Peptoniphilus* | 0.000 | 0.030 | 0.000 |
| *Peptostreptococcaceae_noname* | 0.000 | 0.000 | 0.020 |
| *Polaromonas* | 0.000 | 0.000 | 0.096 |
| *Porphyromonas* | 0.000 | 0.000 | 0.019 |
| *Propionibacteriaceae_unclassified* | 0.005 | 0.057 | 0.329 |
| *Propionibacterium* | 0.903 | 0.201 | 11.144 |
| *Pseudomonas* | 35.195 | 12.904 | 13.761 |
| *Pseudonocardia* | 0.000 | 0.000 | 0.132 |
| *Retroviridae_noname* | 0.000 | 0.000 | 0.135 |
| *Rhizobium* | 1.617 | 0.000 | 0.043 |
| *Roseomonas* | 0.000 | 0.000 | 0.175 |
| *Rothia* | 0.000 | 0.309 | 0.149 |
| *Salinicoccus* | 0.000 | 0.000 | 0.039 |
| *Serinicoccus* | 0.000 | 0.274 | 0.444 |
| *Shewanella* | 0.000 | 0.226 | 0.000 |
| *Siphoviridae_noname* | 0.000 | 0.000 | 1.149 |
| *Sphingobacterium* | 3.109 | 0.044 | 0.000 |
| *Sphingobium* | 1.227 | 0.048 | 0.600 |
| *Staphylococcus* | 28.643 | 63.679 | 21.837 |
| *Stenotrophomonas* | 0.084 | 0.185 | 1.690 |
| **Genus** | **Adult patients  (Mean Relative Abundance %)** | **Paediatric patients  (Mean Relative Abundance %)** | **Healthy controls  (Mean Relative Abundance %)** |
| *Streptococcus* | 0.000 | 1.332 | 0.130 |
| *Veillonella* | 0.000 | 0.088 | 0.016 |
| *Verminephrobacter* | 0.000 | 0.248 | 0.614 |
| *Viruses_noname* | 0.000 | 0.000 | 0.207 |
| *Weissella* | 0.000 | 0.000 | 0.005 |

**Table S4B: Mean relative abundances of all species identified in Adult AD patients, Paediatric AD patients and Healthy control individuals**

| **Species** | **Adult patients  (Mean Relative Abundance %)** | **Paediatric patients  (Mean Relative Abundance %)** | **Healthy controls  (Mean Relative Abundance %)** |
| --- | --- | --- | --- |
| *Halococcus_unclassified* | 0.000 | 0.012 | 0.016 |
| *Natronococcus_unclassified* | 0.000 | 0.000 | 0.037 |
| *Actinomyces_odontolyticus* | 0.000 | 0.149 | 0.000 |
| *Actinomyces_oris* | 0.000 | 0.000 | 0.020 |
| *Brevibacterium_mcbrellneri* | 0.000 | 0.000 | 0.001 |
| *Brevibacterium_unclassified* | 1.800 | 1.604 | 0.293 |
| *Corynebacterium_accolens* | 0.011 | 0.000 | 0.528 |
| *Corynebacterium_ammoniagenes* | 0.000 | 0.000 | 0.012 |
| *Corynebacterium_amycolatum* | 0.005 | 0.000 | 0.000 |
| *Corynebacterium_genitalium* | 0.000 | 0.000 | 0.047 |
| *Corynebacterium_glucuronolyticum* | 0.000 | 0.000 | 0.030 |
| *Corynebacterium_jeikeium* | 0.033 | 0.000 | 0.116 |
| *Corynebacterium_kroppenstedtii* | 0.000 | 0.000 | 0.010 |
| *Corynebacterium_lipophiloflavum* | 0.029 | 0.000 | 2.159 |
| *Corynebacterium_pseudogenitalium* | 0.289 | 0.056 | 0.776 |
| *Corynebacterium_pyruviciproducens* | 0.000 | 0.000 | 0.001 |
| *Corynebacterium_resistens* | 0.000 | 0.378 | 0.262 |
| *Corynebacterium_striatum* | 0.002 | 0.265 | 0.000 |
| *Corynebacterium_tuberculostearicum* | 0.130 | 0.070 | 0.992 |
| *Brachybacterium_muris* | 0.000 | 0.000 | 0.016 |
| *Brachybacterium_paraconglomeratum* | 0.000 | 0.036 | 0.063 |
| *Brachybacterium_squillarum* | 0.000 | 0.000 | 0.000 |
| *Brachybacterium_unclassified* | 0.007 | 1.121 | 1.226 |
| *Kytococcus_sedentarius* | 0.000 | 0.160 | 0.484 |
| *Dietzia_cinnamea* | 0.000 | 0.000 | 0.001 |
| *Dietzia_unclassified* | 0.099 | 0.000 | 2.116 |
| *Gordonia_terrae* | 0.000 | 0.000 | 0.014 |
| *Janibacter_hoylei* | 0.000 | 0.560 | 0.383 |
| *Serinicoccus_marinus* | 0.000 | 0.000 | 0.010 |
| *Serinicoccus_profundi* | 0.000 | 0.000 | 0.041 |
| *Serinicoccus_unclassified* | 0.000 | 0.274 | 0.392 |
| **Species** | **Adult patients  (Mean Relative Abundance %)** | **Paediatric patients  (Mean Relative Abundance %)** | **Healthy controls  (Mean Relative Abundance %)** |
| *Kocuria_rhizophila* | 0.000 | 0.000 | 0.016 |
| *Kocuria_unclassified* | 0.000 | 0.082 | 0.000 |
| *Micrococcus_luteus* | 3.816 | 5.185 | 9.163 |
| *Rothia_aeria* | 0.000 | 0.000 | 0.002 |
| *Rothia_dentocariosa* | 0.000 | 0.026 | 0.049 |
| *Rothia_mucilaginosa* | 0.000 | 0.282 | 0.097 |
| *Rothia_unclassified* | 0.000 | 0.000 | 0.002 |
| *Nocardioides_unclassified* | 0.000 | 0.000 | 0.071 |
| *Propionibacterium_acnes* | 0.903 | 0.198 | 10.837 |
| *Propionibacterium_avidum* | 0.000 | 0.000 | 0.059 |
| *Propionibacterium_granulosum* | 0.000 | 0.003 | 0.248 |
| *Pseudonocardia_unclassified* | 0.000 | 0.000 | 0.132 |
| *Gardnerella_vaginalis* | 0.000 | 0.317 | 0.004 |
| *Atopobium_vaginae* | 0.000 | 0.086 | 0.003 |
| *Porphyromonas_bennonis* | 0.000 | 0.000 | 0.012 |
| *Porphyromonas_gingivalis* | 0.000 | 0.000 | 0.007 |
| *Alloprevotella_unclassified* | 0.000 | 0.000 | 0.005 |
| *Sphingobacterium_unclassified* | 3.109 | 0.044 | 0.000 |
| *Microcoleus_unclassified* | 0.000 | 0.000 | 0.051 |
| *Chroococcidiopsis_thermalis* | 0.000 | 0.000 | 0.174 |
| *Deinococcus_proteolyticus* | 0.000 | 0.000 | 0.005 |
| *Deinococcus_unclassified* | 1.020 | 0.532 | 0.312 |
| *Deinococcus_wulumuqiensis* | 0.000 | 0.000 | 0.035 |
| *Bacillus_megaterium* | 0.000 | 0.000 | 0.032 |
| *Marinococcus_halotolerans* | 0.000 | 0.000 | 0.079 |
| *Oceanobacillus_unclassified* | 0.000 | 0.000 | 0.009 |
| *Exiguobacterium_pavilionensis* | 0.000 | 0.000 | 0.009 |
| *Exiguobacterium_unclassified* | 0.000 | 0.000 | 0.027 |
| *Gemella_haemolysans* | 0.000 | 0.000 | 0.003 |
| *Gemella_unclassified* | 0.000 | 0.000 | 0.005 |
| *Salinicoccus_carnicancri* | 0.000 | 0.000 | 0.039 |
| *Staphylococcus_arlettae* | 0.011 | 2.114 | 0.752 |
| *Staphylococcus_aureus* | 22.171 | 32.861 | 0.000 |
| *Staphylococcus_caprae_capitis* | 0.104 | 0.000 | 1.018 |
| *Staphylococcus_epidermidis* | 2.493 | 16.370 | 2.764 |
| *Staphylococcus_haemolyticus* | 3.557 | 0.400 | 0.480 |
| *Staphylococcus_hominis* | 0.199 | 7.327 | 16.433 |
| *Staphylococcus_lugdunensis* | 0.108 | 0.000 | 0.000 |
| *Staphylococcus_massiliensis* | 0.000 | 0.000 | 0.169 |
| *Staphylococcus_pettenkoferi* | 0.000 | 0.000 | 0.023 |
| *Staphylococcus_saprophyticus* | 0.000 | 0.000 | 0.198 |
| *Staphylococcus_vitulinus* | 0.000 | 4.607 | 0.000 |
| **Species** | **Adult patients  (Mean Relative Abundance %)** | **Paediatric patients  (Mean Relative Abundance %)** | **Healthy controls  (Mean Relative Abundance %)** |
| *Aerococcus_viridans* | 0.001 | 0.000 | 0.058 |
| *Facklamia_languida* | 0.000 | 0.000 | 0.003 |
| *Granulicatella_unclassified* | 0.000 | 0.130 | 0.011 |
| *Weissella_paramesenteroides* | 0.000 | 0.000 | 0.005 |
| *Streptococcus_anginosus* | 0.000 | 0.336 | 0.000 |
| *Streptococcus_australis* | 0.000 | 0.000 | 0.000 |
| *Streptococcus_dysgalactiae* | 0.000 | 0.261 | 0.000 |
| *Streptococcus_infantarius* | 0.000 | 0.098 | 0.000 |
| *Streptococcus_mitis_oralis_pneumoniae* | 0.000 | 0.197 | 0.100 |
| *Streptococcus_mutans* | 0.000 | 0.120 | 0.000 |
| *Streptococcus_pyogenes* | 0.000 | 0.000 | 0.005 |
| *Streptococcus_salivarius* | 0.000 | 0.034 | 0.000 |
| *Streptococcus_sanguinis* | 0.000 | 0.286 | 0.024 |
| *Finegoldia_magna* | 0.000 | 0.234 | 0.000 |
| *Peptoniphilus_harei* | 0.000 | 0.030 | 0.000 |
| *Peptoniphilus_rhinitidis* | 0.000 | 0.000 | 0.000 |
| *Peptostreptococcaceae_noname_unclassified* | 0.000 | 0.000 | 0.020 |
| *Veillonella_parvula* | 0.000 | 0.025 | 0.001 |
| *Veillonella_unclassified* | 0.000 | 0.063 | 0.015 |
| *Asticcacaulis_unclassified* | 0.000 | 0.008 | 0.031 |
| *Brevundimonas_diminuta* | 0.000 | 0.640 | 0.204 |
| *Brevundimonas_unclassified* | 0.472 | 1.476 | 5.058 |
| *Brucella_ovis* | 0.000 | 0.004 | 0.000 |
| *Brucella_pinnipedialis* | 0.033 | 0.784 | 0.003 |
| *Ochrobactrum_intermedium* | 0.782 | 0.000 | 0.000 |
| *Agrobacterium_tumefaciens* | 0.443 | 0.138 | 0.059 |
| *Agrobacterium_unclassified* | 2.577 | 0.014 | 0.107 |
| *Rhizobium_lupini* | 1.617 | 0.000 | 0.043 |
| *Paracoccus_unclassified* | 0.000 | 0.253 | 0.383 |
| *Roseomonas_unclassified* | 0.000 | 0.000 | 0.175 |
| *Sphingobium_unclassified* | 0.000 | 0.048 | 0.000 |
| *Sphingobium_yanoikuyae* | 1.227 | 0.000 | 0.600 |
| *Achromobacter_piechaudii* | 0.000 | 0.106 | 0.000 |
| *Achromobacter_unclassified* | 0.000 | 0.021 | 0.000 |
| *Achromobacter_xylosoxidans* | 0.000 | 0.367 | 0.000 |
| *Alcaligenes_unclassified* | 0.000 | 0.294 | 0.000 |
| *Lautropia_mirabilis* | 0.000 | 0.013 | 0.000 |
| *Comamonas_unclassified* | 0.010 | 0.200 | 0.277 |
| *Delftia_acidovorans* | 1.213 | 0.040 | 0.902 |
| *Delftia_unclassified* | 1.078 | 0.100 | 0.684 |
| *Polaromonas_unclassified* | 0.000 | 0.000 | 0.096 |
| *Verminephrobacter_unclassified* | 0.000 | 0.248 | 0.614 |
| **Species** | **Adult patients  (Mean Relative Abundance %)** | **Paediatric patients  (Mean Relative Abundance %)** | **Healthy controls  (Mean Relative Abundance %)** |
| *Massilia_timonae* | 0.000 | 0.000 | 0.008 |
| *Massilia_unclassified* | 0.000 | 0.062 | 0.043 |
| *Neisseria_meningitidis* | 0.000 | 0.000 | 0.003 |
| *Neisseria_sicca* | 0.000 | 0.000 | 0.003 |
| *Neisseria_unclassified* | 0.000 | 1.060 | 0.072 |
| *Alishewanella_agri* | 0.000 | 0.000 | 0.010 |
| *Shewanella_unclassified* | 0.000 | 0.226 | 0.000 |
| *Klebsiella_pneumoniae* | 0.000 | 0.000 | 0.006 |
| *Klebsiella_unclassified* | 0.000 | 0.000 | 0.011 |
| *Pantoea_dispersa* | 0.000 | 0.000 | 0.002 |
| *Halomonas_stevensii* | 0.000 | 0.000 | 0.019 |
| *Halomonas_unclassified* | 0.000 | 0.000 | 0.050 |
| *Haemophilus_parainfluenzae* | 0.000 | 0.000 | 0.015 |
| *Acinetobacter_baumannii* | 0.000 | 0.000 | 0.001 |
| *Acinetobacter_guillouiae* | 0.000 | 0.017 | 0.000 |
| *Acinetobacter_indicus* | 0.531 | 0.000 | 0.116 |
| *Acinetobacter_johnsonii* | 0.000 | 0.000 | 0.003 |
| *Acinetobacter_junii* | 0.570 | 2.000 | 0.382 |
| *Acinetobacter_lwoffii* | 0.085 | 0.000 | 5.056 |
| *Acinetobacter_pittii* | 0.004 | 0.000 | 0.000 |
| *Acinetobacter_schindleri* | 0.000 | 0.000 | 0.079 |
| *Acinetobacter_unclassified* | 0.163 | 0.000 | 0.252 |
| *Enhydrobacter_aerosaccus* | 13.406 | 0.000 | 1.775 |
| *Pseudomonas_aeruginosa* | 0.000 | 5.426 | 0.000 |
| *Pseudomonas_fragi* | 0.000 | 0.002 | 0.000 |
| *Pseudomonas_psychrotolerans* | 0.000 | 0.651 | 0.035 |
| *Pseudomonas_putida* | 6.121 | 0.000 | 0.161 |
| *Pseudomonas_sp_UK4* | 5.734 | 1.953 | 0.000 |
| *Pseudomonas_stutzeri* | 9.036 | 1.034 | 11.660 |
| *Pseudomonas_synxantha* | 0.000 | 0.000 | 0.000 |
| *Pseudomonas_unclassified* | 14.303 | 3.836 | 1.906 |
| *Stenotrophomonas_maltophilia* | 0.080 | 0.185 | 1.355 |
| *Stenotrophomonas_unclassified* | 0.003 | 0.000 | 0.335 |
| *Penicillium_chrysogenum* | 0.000 | 1.485 | 1.378 |
| *Debaryomyces_hansenii* | 0.000 | 0.047 | 0.000 |
| *Malassezia_globosa* | 0.005 | 0.009 | 8.893 |
| *Ahjdlikevirus_unclassified* | 0.000 | 0.000 | 0.039 |
| *C2likevirus_unclassified* | 0.000 | 0.018 | 0.000 |
| *Propionibacterium_phage_P14_4* | 0.000 | 0.000 | 1.149 |
| *Betapapillomavirus_3* | 0.000 | 0.000 | 0.091 |
| *Human_papillomavirus* | 0.595 | 0.000 | 0.000 |
| *Avian_myelocytomatosis_virus* | 0.000 | 0.000 | 0.039 |
| **Species** | **Adult patients  (Mean Relative Abundance %)** | **Paediatric patients  (Mean Relative Abundance %)** | **Healthy controls  (Mean Relative Abundance %)** |
| *Porcine_type_C_oncovirus* | 0.000 | 0.000 | 0.002 |
| *Avian_endogenous_retrovirus_EAV_HP* | 0.000 | 0.000 | 0.135 |
| *Cuban_alphasatellite_1* | 0.000 | 0.000 | 0.207 |

**Table S5: Number of Gram positive cocci (GPC) isolates in AD patients and Healthy controls**

| **No. of GPCs/individual** | **Patient ID** | **Healthy Control ID** |
| --- | --- | --- |
| 3 | AD003, AD014 | N.A. |
| 2 | AD005, AD006,AD007, AD012,AD013, AD017,  AD025, AD029,AD030, AD031,AD001, AD008,  AD011, AD015,AD033 | AD_9A, AD_9D,  AD_27B |
| 1 | AD009, AD022,AD024, AD028,AD010, AD019,  AD020, AD021,AD032, AD034 | AD_3D, AD_3E, AD_3F, AD_5B, AD_5D, AD_6A,  AD_7A, AD_7C, AD_7D, AD_9B, AD_9C, AD_12A,  AD_12D, AD_12E, AD_13A,  AD_13B, AD_13E, AD_18A,  AD_18B, AD_18C, AD_22A,  AD_22B, AD_22C, AD_24C,  AD_25A, AD_25B, AD_25C,  AD_25D, AD_27A, AD_28A,  AD_28B, AD_29A, AD_30A |
| None | AD016, AD018, AD027, AD002,AD004, AD023,  AD026 | AD_5A, AD_5C, AD_6B,  AD_6C, AD_7B, AD_12B, AD_12C, AD_16A, AD_16B,  AD_16C, AD_17A, AD_17B,  AD_17C, AD_24A, AD_24B,  AD_27C, AD_28C, AD_30B |

**Table S6A: Differentially abundant microbial pathways in AD lesional skin**

| **KEGG Ortholog ID** | **# Pathway** | **Average in Cases (cpm*)** | **Average in Controls (cpm)** |
| --- | --- | --- | --- |
| KO00361 | Chlorocyclohexane and chlorobenzene degradation | 30.82299 | 3.509052 |
| KO00627 | Aminobenzoate degradation | 60.06494 | 26.23713 |
| KO00330 | Arginine and proline metabolism | 120.6917 | 75.61271 |
| KO00643 | Styrene degradation | 58.66755 | 2.640352 |
| KO00121 | Secondary bile acid biosynthesis | 40.01885 | 11.44492 |
| KO05150 | Staphylococcus aureus infection | 35.43843 | 2.235511 |
| KO00440 | Phosphonate and phosphinate metabolism | 22.13243 | 3.42764 |
| KO00310 | Lysine degradation | 112.3122 | 43.53333 |
| KO02010 | ABC transporters | 126.45 | 66.03669 |
| KO00340 | Histidine metabolism | 183.0606 | 89.8119 |
| KO00281 | Geraniol degradation | 136.3255 | 9.586184 |
| KO00780 | Biotin metabolism | 150.5899 | 88.54037 |
| KO00473 | D-Alanine metabolism | 184.049 | 82.6067 |

**Table S6B. Differentially abundant microbial pathways in Healthy control skin**

| **KEGG Ortholog ID** | **# Pathway** | **Average in Cases (cpm*)** | **Average in Controls (cpm)** |
| --- | --- | --- | --- |
| KO00020 | Citrate cycle (TCA cycle) | 69.48878 | 114.7584 |
| KO00290 | Valine, leucine and isoleucine biosynthesis | 227.5606 | 269.8944 |
| KO01051 | Biosynthesis of ansamycins | 181.5392 | 142.784 |
| KO00970 | Aminoacyl-tRNA biosynthesis | 120.7703 | 151.2063 |
| KO00250 | Alanine, aspartate and glutamate metabolism | 90.44557 | 98.63942 |
| KO00471 | D-Glutamine and D-glutamate metabolism | 110.594 | 131.8195 |
| KO00710 | Carbon fixation in photosynthetic organisms | 8.410336 | 34.67165 |
| KO03420 | Nucleotide excision repair | 56.6189 | 77.52548 |
| KO03020 | RNA polymerase | 68.17202 | 71.02269 |
| KO03018 | RNA degradation | 55.82259 | 72.63678 |
| KO04146 | Peroxisome | 6.368264 | 26.50667 |
| KO03010 | Ribosome | 59.17284 | 84.55135 |
| KO03050 | Proteasome | 0.100858 | 8.94913 |

**Table S7. Percentage of AD patients and Healthy controls harbouring at least one AD associated *FLG* LoF in (a) Our study, (b) Indian samples from GenomeAsia 100K and (c) European AD study**

| ***FLG* LoFs globally associated with AD** | **Individuals carrying at least one AD associated *FLG* LoF (%)** | | | | |
| --- | --- | --- | --- | --- | --- |
|  | **Our study** | | **Indian samples (GA100K)**  ([Wall et al., 2019](#_ENREF_8)) | **Europe**  ([Sandilands et al., 2007](#_ENREF_7)) | |
|  | Case  (n=23) | Control  (n=31) | Control  (n=533) | Case  (n=188) | Control  (n=736) |
| 2282del4 | 8.6% (2/23) | 0 | 0.6% (3/533) | 19% (36/188) | 2.6% (19/736) |
| R501X | 0 | 0 | 0 | 27% (51/188) | 2.6% (19/736) |
| 3321delA | 0 | 0 | 0 | 0 | 0 |
| S2554X | 0 | 0 | 0 | 0 | 0 |
| S3316X | 0 | 0 | 0 | 0 | 0 |
| R826X | 0 | 0 | 0 | 0 | 0 |

Figure with Title and Legends:

Figure S1: The levels of MIP-1α among Adult AD patients, Paediatric AD patients and Healthy controls

Legends: Serum cytokine assays were performed using Human Cytokine Pro 27-Plex Assay kit that includes both Th1 and Th2 cytokines. Levels of MIP-1α was found to be positively correlated with disease severity score (EASI). The mean value of MIP-1α in Adult patients was similar to Paediatric patients but higher than the Adult Healthy controls, although not significant. The horizontal line denotes the mean value.

Figure S2: Correlation plot of the summed damaging allele dosage and relative abundance of *Staphylococcus aureus*

Legends: Spearman’s rank correlation between the core microbiome abundance and summed damaging allele dosage of the 5 “potentially damaging” SNPs revealed that species *S. aureus* was significantly negatively correlated (rho=-0.47, p=0.004) with summed damaging allele dosage. “F” is the probability of the slope being not equals to zero.

Figure S3: Correlation plot of the summed damaging allele dosage and relative abundance of order Pseudomonadales

Legends: Spearman’s rank correlation between the core microbiome abundance and summed damaging allele dosage of the 5 damaging SNPs revealed that order Pseudomonadales was significantly positively correlated (rho=-0.47, p=0.02) with summed damaging allele dosage. “F” is the probability of the slope being not equals to zero.

**References:**

Baud, M. (1993). *Data analysis, mathematical modeling.*

Bhattacharyya, C., Majumder, P.P., and Pandit, B. (2018). CXCL10 is overexpressed in active tuberculosis patients compared to M. tuberculosis-exposed household contacts. *Tuberculosis* 109**,** 8-16.

Chopra, R., Vakharia, P.P., Sacotte, R., Patel, N., Immaneni, S., White, T., et al. (2017). Severity strata for Eczema Area and Severity Index (EASI), modified EASI, Scoring Atopic Dermatitis (SCORAD), objective SCORAD, Atopic Dermatitis Severity Index and body surface area in adolescents and adults with atopic dermatitis. *British Journal of Dermatology* 177**,** 1316-1321.

Klymiuk, I., Bambach, I., Patra, V., Trajanoski, S., and Wolf, P. (2016). 16S Based Microbiome Analysis from Healthy Subjects’ Skin Swabs Stored for Different Storage Periods Reveal Phylum to Genus Level Changes. *Frontiers in Microbiology* 7.

On, H.R., Lee, S.E., Kim, S.E., Hong, W.J., Kim, H.J., Nomura, T., et al. (2017). Filaggrin Mutation in Korean Patients with Atopic Dermatitis. *Yonsei medical journal* 58**,** 395-400.

Robin, J.D., Ludlow, A.T., Laranger, R., Wright, W.E., and Shay, J.W. (2016). Comparison of DNA Quantification Methods for Next Generation Sequencing. *Scientific Reports* 6**,** 24067.

Sandilands, A., Terron-Kwiatkowski, A., Hull, P.R., O'regan, G.M., Clayton, T.H., Watson, R.M., et al. (2007). Comprehensive analysis of the gene encoding filaggrin uncovers prevalent and rare mutations in ichthyosis vulgaris and atopic eczema. *Nature Genetics* 39**,** 650.

Wall, J.D., Stawiski, E.W., Ratan, A., Kim, H.L., Kim, C., Gupta, R., et al. (2019). The GenomeAsia 100K Project enables genetic discoveries across Asia. *Nature* 576**,** 106-111.
