# Supplementary figures and images for: Dysbiotic Lesional Microbiome With Filaggrin Missense Variants Associate With Atopic Dermatitis in India
Source: Front Cell Infect Microbiol. 2020 Nov 17;10:570423. doi: 10.3389/fcimb.2020.570423 (PMC7705349; doi:10.3389/fcimb.2020.570423)

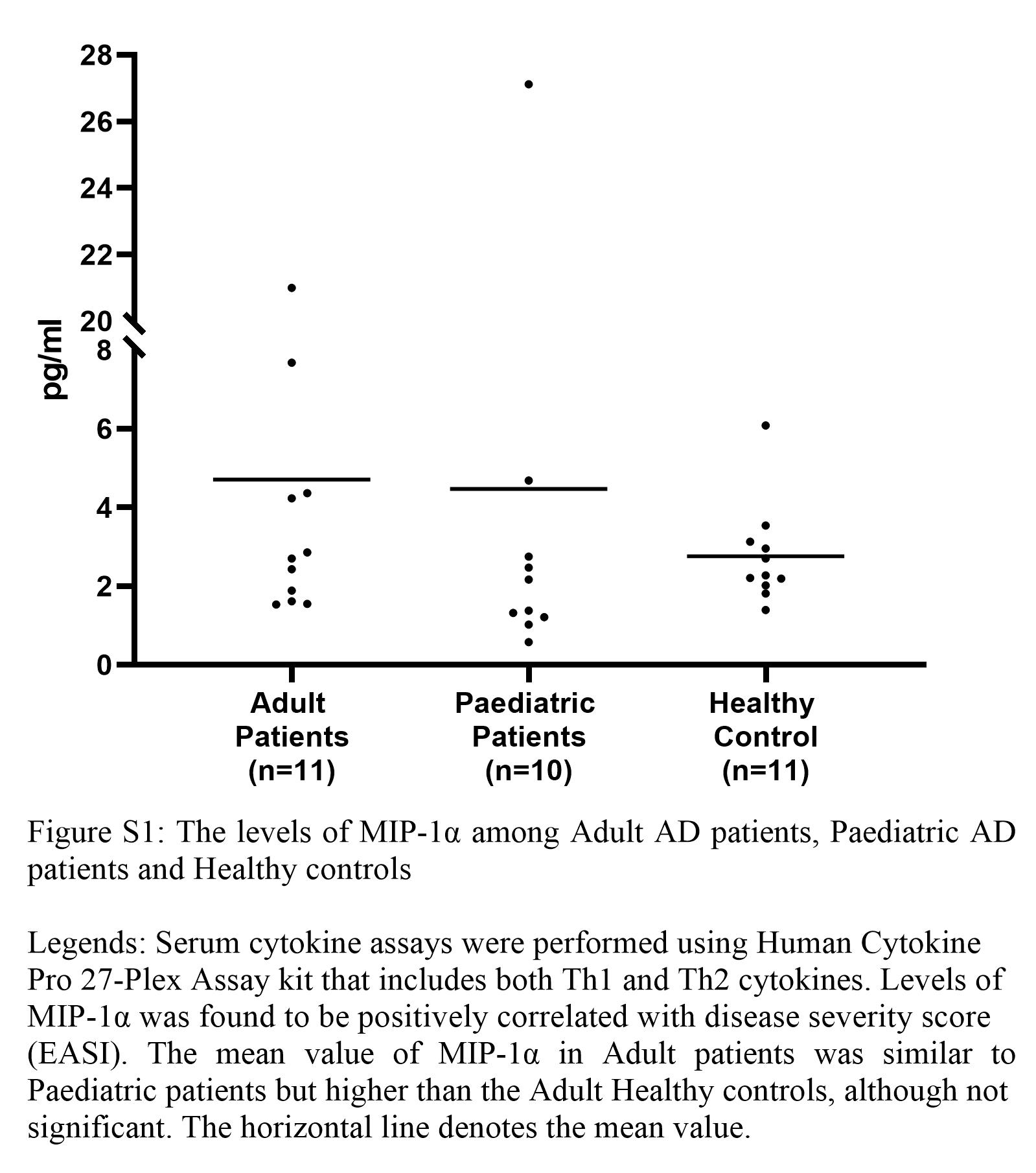

Supplement: Supplementary file 2 [file Image_1.jpg]

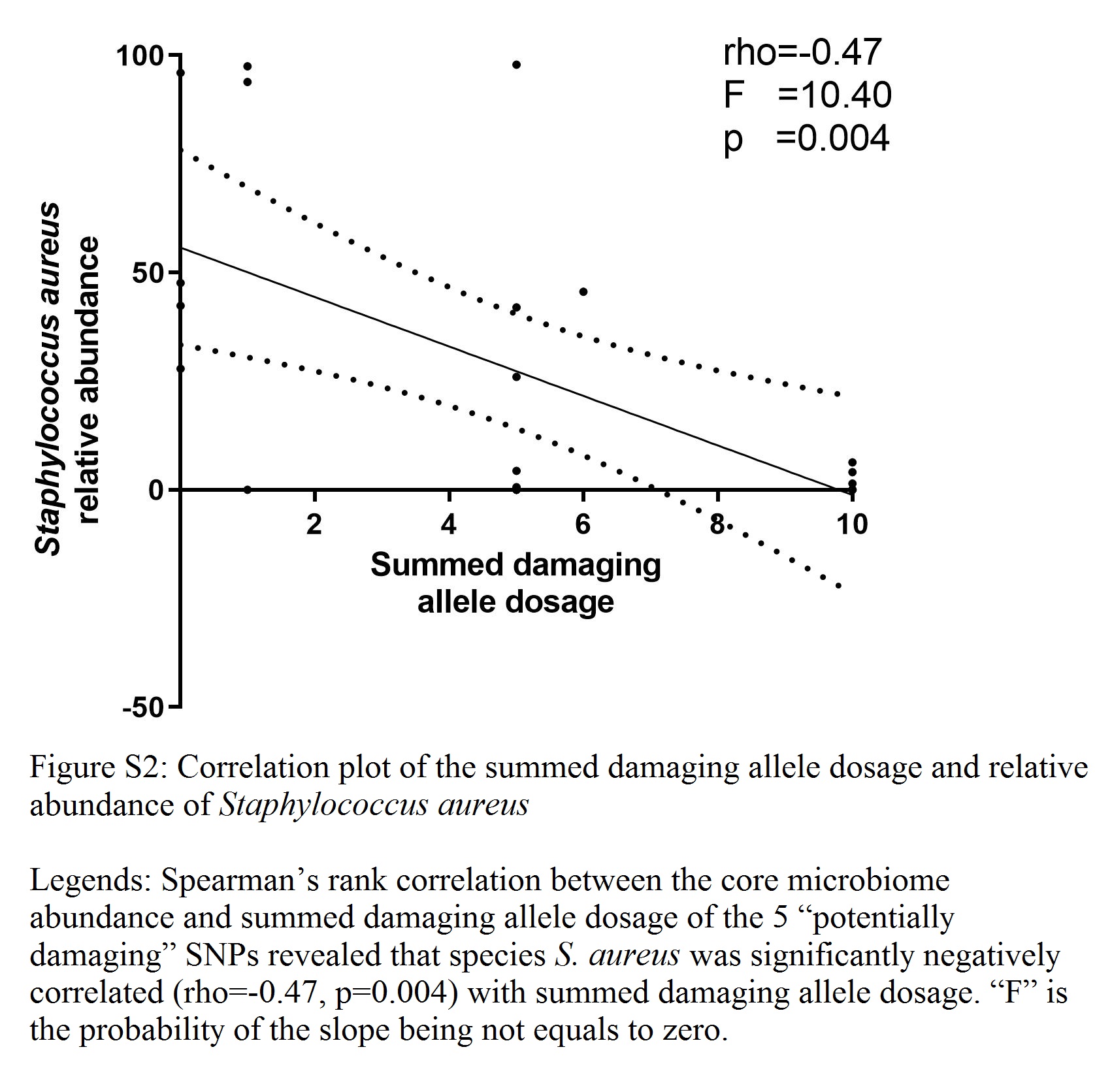

Supplement: Supplementary file 3 [file Image_2.jpg]

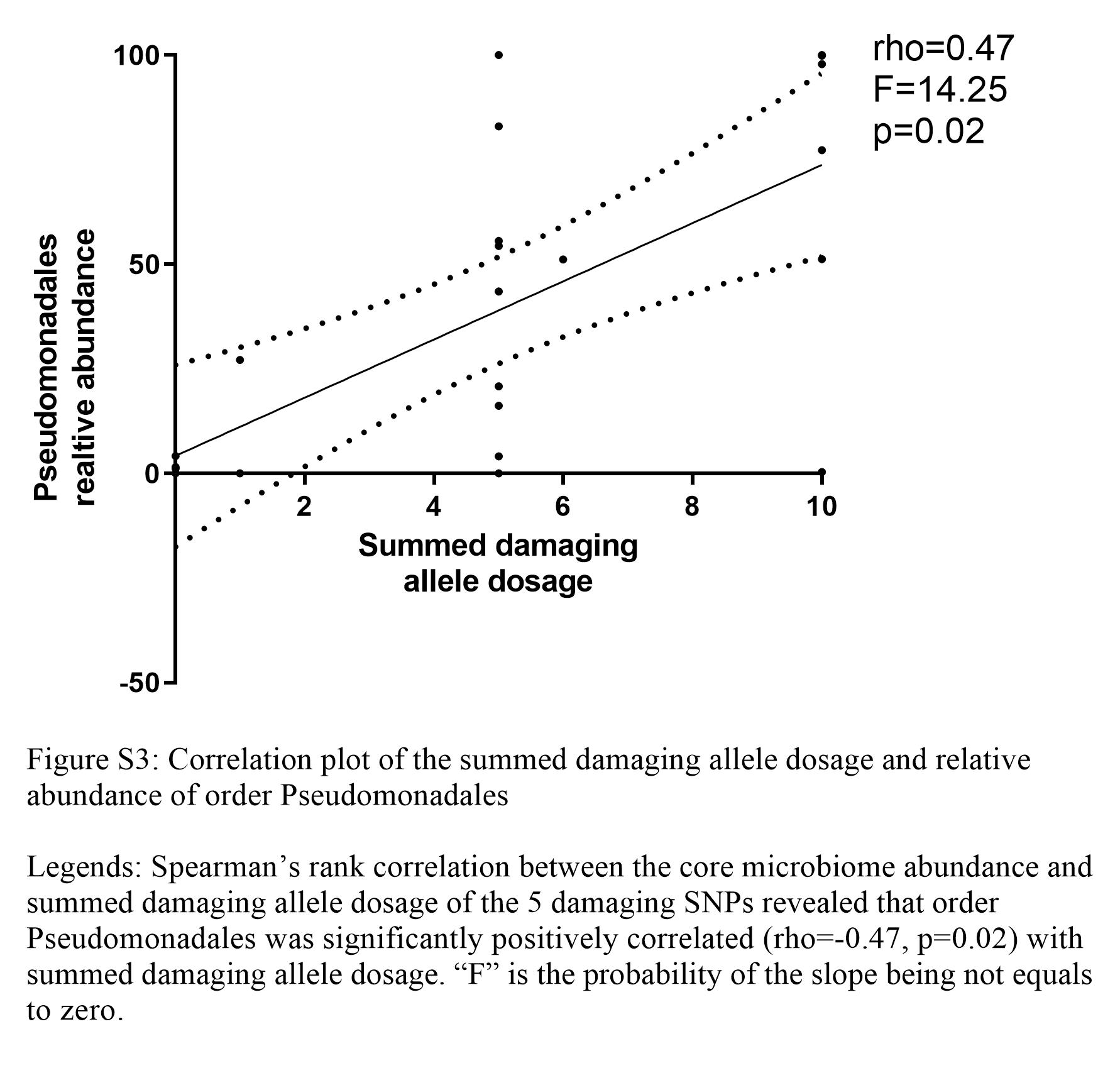

Supplement: Supplementary file 4 [file Image_3.jpg]
